# AMPK promotes antitumor immunity by downregulating PD-1 in regulatory T cells via the HMGCR/p38 signaling pathway

**DOI:** 10.1186/s12943-021-01420-9

**Published:** 2021-10-14

**Authors:** Ram Hari Pokhrel, Suman Acharya, Jae-Hee Ahn, Ye Gu, Mahesh Pandit, Jong-Oh Kim, Yun-Yong Park, Ben Kang, Hyun-Jeong Ko, Jae-Hoon Chang

**Affiliations:** 1 College of Pharmacy, Yeungnam University, 280 Daehak-ro, Gyeongsan-si, Gyeongbuk-do 38541 Republic of Korea; 2 Department of Pharmacy, Kangwon National University, Kangwondaehak-gil 1, Chuncheon, 24341 Republic of Korea; 3 Department of Life Science, Chung-Ang University, Seoul, 06974 Republic of Korea; 4 Department of Pediatrics, School of Medicine, Kyungpook National University, Daegu, 41944 Republic of Korea

**Keywords:** Tregs, Tumor, PD-1, AMPK, HMGCR, Compound C, AICAR

## Abstract

**Background:**

AMP-activated protein kinase (AMPK) is a metabolic sensor that maintains energy homeostasis. AMPK functions as a tumor suppressor in different cancers; however, its role in regulating antitumor immunity, particularly the function of regulatory T cells (Tregs), is poorly defined.

**Methods:**

*AMPKα1*^fl/fl^*Foxp3*^YFP-Cre^, *Foxp3*^YFP-Cre^, *Rag1*^*−/−*^, and C57BL/6 J mice were used for our research. Flow cytometry and cell sorting, western blotting, immuno-precipitation, immuno-fluorescence, glycolysis assay, and qRT-PCR were used to investigate the role of AMPK in suppressing programmed cell death 1 (PD-1) expression and for mechanistic investigation.

**Results:**

The deletion of the AMPKα1 subunit in Tregs accelerates tumor growth by increasing the expression of PD-1. Metabolically, loss of AMPK in Tregs promotes glycolysis and the expression of 3-hydroxy-3-methylglutaryl-CoA reductase (HMGCR), a key enzyme of the mevalonate pathway. Mechanistically, AMPK activates the p38 mitogen-activated protein kinase (MAPK) that phosphorylates glycogen synthase kinase-3β (GSK-3β), inhibiting the expression of PD-1 in Tregs.

**Conclusion:**

Our study identified an AMPK regulatory mechanism of PD-1 expression via the HMGCR/p38 MAPK/GSK3β signaling pathway. We propose that the AMPK activator can display synergic antitumor effect in murine tumor models, supporting their potential clinical use when combined with anti-PD-1 antibody, anti-CTLA-4 antibody, or a HMGCR inhibitor.

**Supplementary Information:**

The online version contains supplementary material available at 10.1186/s12943-021-01420-9.

## Background

Tumor cells reprogram several metabolic pathways to meet their bioenergetics/biosynthetic demands [1]; this leads to changes in the tumor microenvironment (TME), affecting tumor-infiltrating cells [2]. Regulatory T cells (Tregs) are a major barrier to antitumor immunity [3]. In the TME, Tregs also undergo metabolic reprogramming, where glycolysis is inhibited, but fatty acid oxidation (FAO) and oxidative phosphorylation are promoted, thereby enhancing Treg-mediated immunosuppression and promoting tumor progression [4]. However, contrary to Tregs, cytotoxic CD8^+^ T cells play an important role in antitumor immunity through the production of interferon (IFN)-γ and granzyme B (GZB) [5]. Notably, T cell activation also depends on metabolic pathways, including aerobic glycolysis, amino acid metabolism, glutaminolysis, and de novo fatty acid synthesis [6]. Since these pathways are equally important for the proliferation and survival of tumor cells, T cells and tumor cells compete for nutrients. Therefore, cellular metabolism actively regulates tumorigenesis.

AMP-activated protein kinase (AMPK) is an evolutionarily conserved serine/threonine kinase acting as an energy sensor for the maintenance of energy homeostasis [7]. AMPK activation by the tumor suppressor Liver kinase B1 (LKB1) supports the hypothesis that AMPK is also a tumor suppressor [8]. Recent studies have shown that AMPK mediates the inhibition of cell proliferation and growth of tumor cells [9]. Notably, studies have shown that the AMPK activators metformin and 5-aminoimidazole-4-carboxamide 1-β-D-ribofuranoside (AICAR) inhibit tumor progression [10, 11]. AMPK also inhibits the expression of Glut1 and glycolysis in Tregs by inhibiting mTORC1 signaling [12]. As glycolysis and lipid metabolism are the primary pathways underpinning the survival of tumor cells, we can speculate that AMPK and Tregs are metabolically linked in cancer. However, the potential role of AMPK in Tregs in cancer has not been studied yet.

Programmed cell death 1 (PD-1) is an inhibitory molecule expressed on the surface of Tregs and effector T cells that serves as a major immune checkpoint in antitumor responses [13]. In fact, anti-PD-1-mediated immune checkpoint blockade is used as first-line therapy against lung cancer [14]. Interestingly, a recent study showed that the inhibition of glycogen synthase kinase-3 beta (GSK3β) decreases the expression of PD-1 on CD8^+^ T cells and is as effective as anti-PD-1/anti-PD-L1 antibodies for controlling B16F10 melanoma [15]. Thus, although the regulatory mechanism of PD-1 expression in Tregs is unclear, GSK3β could serve as a potential target for the modulation of PD-1 in cancer.

In the present study, we addressed the role of AMPK in Tregs in cancer by generating Treg-specific AMPKα1-knockout mice. We showed that AMPK loss in Treg cells accelerated tumor growth by increasing the expression of PD-1. Our results indicate that AMPK regulates the expression of PD-1 via the HMGCR/P38 MAPK/GSK3β signaling axis, suppressing tumor progression.

## Materials and methods

### Mice

C57BL/6 J, *Rag1*^*−/−*^*, AMPKα1*^fl/fl^, *CD4*^Cre^ and *Foxp3*^YFP-Cre^ mice were purchased from the Jackson Laboratory (Bar Harbor, ME, USA). *AMPKα1*^fl/fl^ mice were crossed with *Foxp3*^*YFP-Cre*^ mice or *CD4*^Cre^ to generate *AMPKα1*^fl/fl^*Foxp3*^YFP-Cre^ offspring (referred to as *AMPK*^fl/fl^*Foxp3*-Cre mice hereafter or also denoted as AMPK-KO in figure) or *AMPKα1*^fl/fl^*CD4*^Cre^ offspring (referred to as *AMPK*^fl/fl^*CD4*-Cre mice hereafter) respectively. *AMPKα1*^fl/fl^*Foxp3*^YFP-Cre^ mice were used at 6–10 weeks of age unless otherwise specified. Age- and sex-matched littermate *Foxp3*^*Cre*^ control mice were used as controls and referred to as wild type (WT) hereafter. All mice were maintained under specific pathogen-free conditions in the animal facilities of the Kangwon National University and the Yeungnam University. All animal experiments were approved by the Institutional Animal Care and Use Committees (IACUC) of the Kangwon National University (Permit Number: KW-190729-1) and the Yeungnam University (Permit Number: 2017–034).

### Tumor models

*AMPKα1*^fl/fl^*Foxp3*^YFP-Cre^ and *Foxp3*^YFP-Cre^, *Rag1*^*−/−*^, and C57BL/6 J mice were injected subcutaneously with 2.5 × 10^5^ B16F10 melanoma cells. WT and *AMPK*^fl/fl^*Foxp3*-Cre mice were injected subcutaneously with 1 × 10^6^ TC-1 cervical cancer cells and 2.5 × 10^5^ MC38 colon cancer cells. Tumors were measured every day with a digital caliper in two dimensions (length and width); tumor volume (mm^3^) was determined using the formula V = W^2^ × L/2, where W and L are the shortest and longest diameters in mm, respectively. Checkpoint blockade monoclonal antibodies were administered every 3 days starting from day 8 post-tumor challenge until the end of the experiment. Anti-mouse PD-1 (Clone#RPM1-14; BioXcell, Lebanon, NH, USA) and anti-mouse cytotoxic T-lymphocyte associated protein 4 (CTLA4) (Clone#9D9; BioXcell) were administered intraperitoneally (i.p.; 200 μg/mouse and 100 μg/mouse, respectively). AICAR (500 mg/kg) was injected i.p. daily either alone or in combination with anti-PD-1 or anti-CTLA4. Statin (15 mg/kg) was injected i.p. daily into C57BL/6 J mice either alone or in combination with AICAR. Mice were assigned into different groups in a randomized fashion based on their ear tag number.

For the isolation of tumor-infiltrating lymphocytes, tumor tissues were harvested and minced using sterile razor blades. Cleared tumor pieces were digested using an enzyme mixture containing 0.5 mg/mL Collagenase D (Cat#11088866001, Roche Dagnostics GmBH, Mannheim, Germany) and 0.02 mg/mL DNase I (Cat#10104159001, Sigma Aldrich, St. Louis, MO, USA) in RPMI 1640 at 37 °C for 45 min and passed through 70 μM cell strainers (BD Biosciences, Franklin Lakes, NJ, USA). A Percoll gradient (Cat#17-0891-01, GE Healthcare, Chicago, IL, USA) was then used to separate cancer cells and enrich lymphocytes as described previously [16].

### Flow cytometry and cell sorting

Single cell suspensions were prepared from spleens, peripheral lymph nodes, and mesenteric lymph nodes. Cells were then lysed on ice with red blood cell lysis solution (Sigma Aldrich, St. Louis, MO, USA), washed with RPMI, and suspended in complete media (RPMI 1640 containing 10% fetal bovine serum and 1% streptomycin and penicillin antibiotics). Fluorescence-labeled anti-CD3 (17A2), anti-CD4 (GK1.5), anti-CD8 (SK1), anti-CD39 (Duha59), anti-CD73 (TY/11.8), anti-ICOS (7E.17G9), anti-PD-1 (RPM1-30), anti-CD304 (Nrp1, 3E12), anti-CD357 (GITR, DTA-1), anti-OX40 (OX-86), anti-IFN-γ (XMG1.2), anti-IL-17 (TC11-18H10.1), anti-T-bet (4B10), and anti-IL-10 (JES5-16E3) antibodies were purchased from Biolegend (San Diego, CA, USA) and used at 1:100 dilution. For intracellular staining, cells were stimulated for 4–6 h with phorbol 12-myristate 13-acetate Protein 3 (50 ng/mL, Sigma Aldrich) plus ionomycin (750 ng/mL, Sigma Aldrich) in the presence of 10 μg/mL Golgistop (BD Biosciences). After incubation, cells were surface-stained with anti-CD4 and anti-CD8 antibodies followed by fixation and permeabilization using a commercial buffer (BD Cytofix/Cytoperm™, BD Biosciences, Franklin Lakes, NJ, USA). For Foxp3 staining, cells were fixed and permeabilized using the Foxp3/Transcription Factor Staining Buffer (eBioscience, Waltham, MA, USA). IFN-γ^+^ Th1 cells, IL-17A^+^ Th17 cells, and CD8^+^GZB^+^ cells were determined by flow cytometry as described previously [17]. YFP^+^CD25^+^ Tregs were sorted using the BD FACSJazz™ cell sorter (BD Biosciences). Flow cytometry data were acquired using a BD FACS Verse flow cytometer (BD Biosciences) and analyzed using the FlowJo software, version 10.2 (FlowJo LLC, Ashland, OR, USA).

### Chemical reagents

C57BL/6 J mice were treated daily with AICAR (2840, 500 mg/kg) purchased from Tocris Bioscience (Abingdon, UK), compound C (Cat#171260, 2.5 mg/kg), GSK-3β inhibitor (Cat#S3442, 200 μg/kg), p38 MAPK inhibitor (Cat#S8307, 2 mg/kg), GGPP (Cat#G3278, 10 mg/kg), mevalonate (Cat#90469, 10 mg/kg), and cholesterol (Cat#C5951, 5 mg/kg) purchased from Sigma Aldrich (St. Louis, USA) and Simvastatin (Cat#10010344, 15mg/kg) purchased from Cayman Chemical Company (Ann Arbor, MI, USA) i.p. To assess the effect of the treatments, mice were euthanized, and single cell suspensions were prepared from their spleens. The expression of several surface markers including PD-1, CTLA4, Nrp1, ICOS, GITR, CD73, CD39, and OX40 was analyzed in Foxp3^+^ Tregs by flow cytometry.

### Western blotting

Sorted YFP^+^CD25^+^ Tregs from WT or *AMPK*^fl/fl^*Foxp3*-Cre mice were stimulated with anti-CD3/CD28 antibodies followed by treatment with AICAR (2 mM), compound C (20 μM), GSK-3β inhibitor (20 μM), or p38 MAPK inhibitor (2 and 5 μM), and were lysed with radio immunoprecipitation buffer containing proteasome inhibitors. The protein concentration was measured via the bicinchoninic acid method. SDS-PAGE was performed as described previously [18]. Immunoblotting was performed using primary antibodies against PD-1 (Cat#BE0146, BioXcell, Lebanon, NH, USA), AMPKα1 (Cat#2795), AMPKα1 Thr172-p (Cat#50081S), HMGCR (Cat#sc-271,595, Santa Cruz Biotechnology, Dallas, TX, USA), Foxo3a Ser253 (Cat#9466), Foxo3a (Clone#D19A7), ACC Ser79 (Clone#D7D11), ACC (Clone#C83B10), SREBP1 (Clone#2A4, Santa Cruz Biotechnology), β-catenin (Cat#8480), β-catenin Ser675 (Cat#11887), GSK-3β (Cat#12456), GSK-3β Ser9 (Cat#5558), LKB1 (Clone#D60C5), p38MAPK Thr180 (Clone#D3F9), p38MAPK (Clone#D13E1), T-bet (Clone#D698B), Erk 1/2 Thr202/Tyr204 (Cat#4370), Erk 1/2 (Cat# 9102), JNK (Cat# 9252), JNK Thr 183/Tyr185 (Cat# 9255) and β-actin (Cat#sc-47778, Santa Cruz Biotechnology). Blots were incubated with primary antibodies at a 1:1000 dilution overnight at 4 °C. On the next day, the blots were incubated with secondary antibodies at 24 °C for 1 h. Protein bands were visualized using a chemiluminescence kit (Cat#34580, Pierce, Appleton, WI, USA).

### Immunoprecipitation

Tregs were harvested from WT mice and lysed in IP lysis buffer (Cat#87787, Pierce, Appleton, WI, USA). Immunoprecipitation was performed using anti-AMPK, anti-p38, anti-GSK3β, and anti-HMGCR antibodies (Cell Signaling Technology) in 200 μL of total cell lysate mixed with 25 μL protein G agarose beads (Cat#22851, Pierce), followed by overnight incubation at 4 °C. The immunoprecipitated proteins were then washed 3–5 times with lysis buffer and analyzed by western blotting.

### Glycolysis assay

YFP^+^CD25^+^ Tregs sorted from WT and AMPK-KO mice were seeded into 96-well plates at a density of 5 × 10^5^ cells/well, followed by incubation overnight in a 5% CO_2_ atmosphere at 37 °C. On the next day, a CO_2_ purge was performed by incubating cells in a CO_2_-free incubator at 37 °C for 3 h. Cell suspensions were then harvested and washed with respiration buffer. The glycolysis assay reagent (Cat#ab197244, Abcam, Cambridge, UK) was then added. Fluorescence intensity was measured at the excitation and emission wavelengths of 380 nm and 615 nm, respectively.

### In vitro Tregs suppression assay

Naïve CD4^+^CD25^−^ T cells were labeled with carboxyfluorescein diacetate succinimidyl ester (CFSE, ThermoFisher Scientific) and co-cultured with an increasing ratio of sorted Tregs for 4 days in the presence of anti-CD3 (1 μg/mL) and irradiated splenocytes. The suppressive activity of Tregs was analyzed by measuring the proliferation of activated effector T cells based on CFSE dilution as described previously [19].

### In vivo cytotoxic T lymphocyte (CTL) activity

To assess CTL responses in vivo, splenocytes were isolated from naïve C57BL/6 mice, divided into equal quantities and stained using a low concentration (0.5 μM) or a high concentration (5 μM) of cell trace violet (Cat#C34571, Invitrogen, Waltham, MA, USA) for 15 min at 37 °C. Then, 5 μM CTV-stained splenocytes were pulsed with 5 μg/mL of E6_41–50_[EVYD-FAFRDL] and E7_49–57_[RAHYNIVTF] peptides (TC-1 tumor-specific epitopes) for 1 h at 37 °C. Afterward, stained cells were mixed at a 1:1 ratio; 2 × 10^7^ cells were then intravenously injected into each mouse. Mice were euthanized 24 h later. Lymphocytes were prepared from the spleen and inguinal lymph nodes and analyzed by flow cytometry. The specific lysis ratio was calculated as r (ratio) = (% CTV^high^ / CTV^low^), and the percent lysis (%) was calculated as lysis % = [1 - (r_unpulsed_ / r_pulsed_)] × 100.

### Immunofluorescence

Paraffin-embedded TC-1 tumor tissues were sliced with a microtome into 4 μm-thick sections; the sections were then placed on slides, deparaffinized, and rehydrated. Antigen retrieval was conducted by microwaving with sodium citrate buffer. Blocking was conducted with PBS containing 1% BSA for 1 h at 20 °C. FITC-conjugated anti-mouse/human GZB antibodies (1:100) and rat anti-mouse CD8 antibodies (1:100) used to probe the sections overnight at 4 °C. Then the sections were incubated with Alexa Fluor 647-conjugated anti-rat IgG antibody for 2 h at 20 °C. Then, 4 ′, 6 ′-diamidino-2-phenylindole was used to counter-stain the nuclei. Stained sections were visualized by confocal microscopy (LSM880 NLO, Carl Zeiss, Jena, Germany).

### RNA isolation and real-time PCR

Total RNA was extracted from sorted YFP^+^CD25^+^ Tregs using the ReliPrep™ RNA Cell Miniprep System (Cat#Z6011, Promega Corporation, Madison, WI, USA), and cDNA was synthesized using the Goscript Reverse Transcription system (Cat#A5001, Promega Corporation). mRNA expression was measured by real-time PCR using the QuantiTect SYBR Green PCR kit (QIAGEN). 25 μL of the mixture, containing 10 ng of the total RNA sample, was used for real-time PCR which was performed on a CFX96 thermal cycler (Bio-Rad, Hercules, CA, USA) using the following thermal cycle: 60 min at 42 °C (for reverse transcription), 15 min at 95 °C (for heat inactivation or pre-denaturation), and 40 cycles for 15 s at 95 °C, 30 s at 58 °C, and 30 s at 72 °C, each. All data analyses were performed using the comparative C_T_ method, and the fold change was calculated using the 2^-△△C(T)^ equation, as previously described [20].

$${2}^{-\triangle \triangle \mathrm{C}\left(\mathrm{T}\right)}=\left[\left({\mathrm{C}}_{\mathrm{T}}\kern0.5em \mathrm{of}\ \mathrm{gene}\ \mathrm{of}\ \mathrm{interest}-{\mathrm{C}}_{\mathrm{T}}\kern0.5em \mathrm{of}\ \mathrm{internal}\ \mathrm{control}\right)\mathrm{Sample}\ \mathrm{A}-\left({\mathrm{C}}_{\mathrm{T}}\kern0.5em \mathrm{of}\ \mathrm{gene}\ \mathrm{of}\ \mathrm{interest}-{\mathrm{C}}_{\mathrm{T}}\kern0.5em \mathrm{of}\ \mathrm{internal}\ \mathrm{control}\right)\mathrm{Sample}\ \mathrm{B}\Big)\right]$$

Melting curve analysis was performed to check for non-specific amplification and to confirm that a single amplicon was generated by qPCR. The PCR efficiency was > 90%. The PCR target genes and primer sequences are listed in supplementary table.

### Statistical analysis

Statistical analysis was performed using GraphPad Prism 9 (GraphPad Software Inc., San Diego, CA, USA). The unpaired two-tailed student’s *t*-test was used for comparisons between two groups. Tukey’s multiple comparisons test was used for multiple comparisons. Data are presented as the mean ± standard deviation (SD). Statistical significance was defined as *p <* 0.05.

## Results

### AMPK deficiency in Tregs promotes tumor growth

**Fig. 1:**
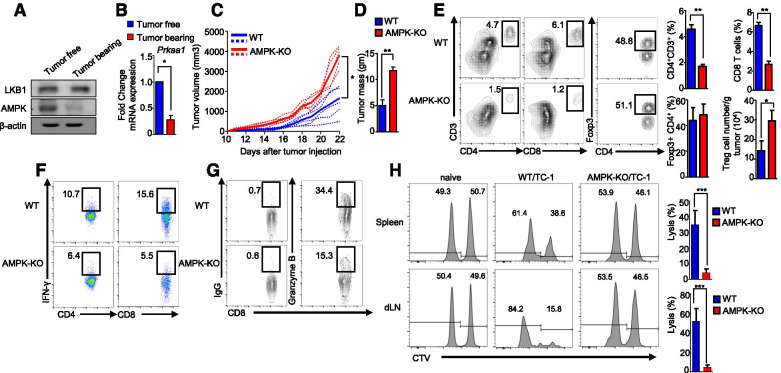
AMPK deficiency in Tregs promotes tumor growth. B16F10 melanoma cells were injected subcutaneously into C57BL/6 J mice and analyzed after 22 days. **A** Analysis of total LKB1 and AMPK in Tregs obtained from tumor-free and tumor-bearing C57BL/6 J mice by western blotting. **B** Detection of the mRNA expression of the *prkaa1* gene by real-time PCR in Tregs isolated from tumor-free and tumor-bearing C57BL/6 J mice. The **C** tumor volume and **D** Tumor weight in WT and *AMPK*^fl/fl^*Foxp3*-Cre mice injected s.c. with B16F10 melanoma cells. **E** Flow cytometric analysis of the percentage of CD4^+^ and CD8^+^ T cells; CD4^+^Foxp3^+^ Tregs and Tregs cellularity in the tumors of WT and *AMPK*^fl/fl^*Foxp3*-Cre mice. **F** Analysis of IFN-γ-producing CD4^+^ and CD8^+^ T cells percentage in tumors from WT and *AMPK*^fl/fl^*Foxp3*-Cre mice by flow cytometry. **G** Flow cytometry analysis of GZB-producing CD8^+^ T cells in tumors from WT and *AMPK*^fl/fl^*Foxp3*-Cre mice. **H** Evaluation of in vivo cytolytic activity in the spleen and draining lymph node of WT and *AMPK*^fl/fl^*Foxp3*-Cre mice. The data are presented as the mean ± standard deviation (SD); *n* = 5 mice per group. *P < 0.05; **P < 0.01; ***P < 0.001

Although the T cell-specific deletion of AMPK promotes tumor growth in mice [21], the specific role of AMPK in Tregs in antitumor immunity is still controversial. Interestingly, we found that the AMPK expression levels were markedly reduced in Tregs from tumor tissues and draining lymph nodes in tumor-bearing mice, compared to those of peripheral Treg cells in tumor-free mice, whereas the levels of LKB1, an upstream regulator of AMPK, remained unchanged (Fig. 1A). Likewise, the transcription of AMPK also decreased in Tregs in tumor-bearing mice, compared to that in WT tumor-free mice (Fig. 1B). To determine whether the correlation between AMPK and PD-1 expression was also evident in cancer patients, we analyzed gene expression profile from public database such as TCGA and GEO [22, 23]. In single-cell RNA-seq dataset which was originated from melanoma tissue (GSE72056), we downloaded the TPM dataset file and sorted any values more than 3 TPM for cells that highly expressed Foxp3 transcripts and excluded invalid TPM values for *Pdcd1* and *Prkaa1* expression. There was a significant negative correlation between the transcripts of Prkaa1 and Pdcd1 at the single-cell level (*r* = − 0.627, *p* = 0.012; Fig. S1A). In addition, the gene expression profiles of *Prkag2* and *Pdcd1* were analyzed in resident Tregs in patient cancer specimens using the available datasets (GSE89225) (*r* = − 0.621, *p* = 0.0034; Fig. S1B). Further analysis also revealed that *Pdcd1* was negatively correlated with AMPK subunit mRNAs including *Prkaa1*, *Prkaa2*, and *Prkag1* in TCGA database obtained from prostate, melanoma, and breast cancer patients (Fig. S1C). These results suggest that Tregs with lower levels of AMPK expression exhibited higher levels of PD-1 expression in patients with cancer, indicating the importance of AMPK in the regulation of PD-1 expression in Tregs.

To address the specific role of AMPK in tumor-associated Tregs, we crossed mice carrying loxP-flanked *Prkaa1* alleles (*AMPK*^fl/fl^) with Foxp3^YFP-Cre^ (referred to as *Foxp3*-Cre) mice [24] to generate progeny in which AMPK alleles are conditionally deleted in Treg cells, but not in other T cells (hereafter referred to as *AMPK*^fl/fl^*Foxp3*-Cre mice; *AMPK*^+/+^*Foxp3*-Cre mice were used as control (WT)). *AMPK*^fl/fl^*Foxp3*-Cre mice (also denoted as AMPK-KO in the figure) were born at the expected Mendelian ratios and seemed grossly normal. There was no differences between the size of the spleen and lymph nodes of WT and *AMPK*^fl/fl^*Foxp3*-Cre mice (data not shown). *AMPK*^fl/fl^*Foxp3*-Cre mice exhibited no significant change in the number and percentage of CD4^+^, CD8^+^ T cells, effector T, Th1, Th17 and Treg cells in spleen, MLN and PLN (Fig. S2A-E). Next, we inoculated several kinds of syngeneic tumor cells into *AMPK*^fl/fl^*Foxp3*-Cre mice and into WT control mice. Interestingly, we found that tumors formed by B16F10 melanoma, TC-1 cervical cancer, and MC38 colon cancer cells grew more rapidly in *AMPK*^fl/fl^*Foxp3*-Cre mice than in WT mice (Fig. 1C, D and Fig. S3A-C).

Moreover, we found that the levels of effector T cells (CD4^+^ and CD8^+^) in the tumors and draining lymph nodes (dLNs) of *AMPK*^fl/fl^*Foxp3*-Cre mice were significantly reduced (Fig. 1E and Fig. S4A). Additionally, although the frequencies of Foxp3^+^ Tregs in the tumors and dLNs were unchanged (Fig. 1E and Fig. S4B), cellularity and proliferation were higher in Tregs from *AMPK*^fl/fl^*Foxp3*-Cre mice (Fig. 1E and Fig. S4C), suggesting that the proliferation and survival of Tregs might be regulated by AMPK. In addition, IFN-γ-producing CD4^+^ and CD8^+^ T cells and GZB-producing CD8^+^ T cells were significantly decreased in tumors and dLNs from *AMPK*^fl/fl^*Foxp3*-Cre mice (Fig. 1F, G, and Fig. S4D, E). In addition, we confirmed that GZB-producing CD8^+^ T cells in tumors were found to be reduced in *AMPK*^fl/fl^*Foxp3*-Cre mice (Fig. S5). Next, we assessed the tumor-antigen-specific cytotoxic T cell response using an in vivo CTL assay, and we observed that cytolytic activity of CD8^+^ T cells in the spleen and lymph nodes was significantly decreased in *AMPK*^fl/fl^*Foxp3*-Cre mice (Fig. 1H), suggesting that AMPK supports the cytotoxic effect exerted by CD8^+^ T cells. Thus, these findings suggest that the loss of AMPK in tumor-associated Tregs promoted tumor growth in mice via the impact on antitumor T cells and their functions.

### AMPK-deficient Tregs show high expression of PD-1

**Fig. 2:**
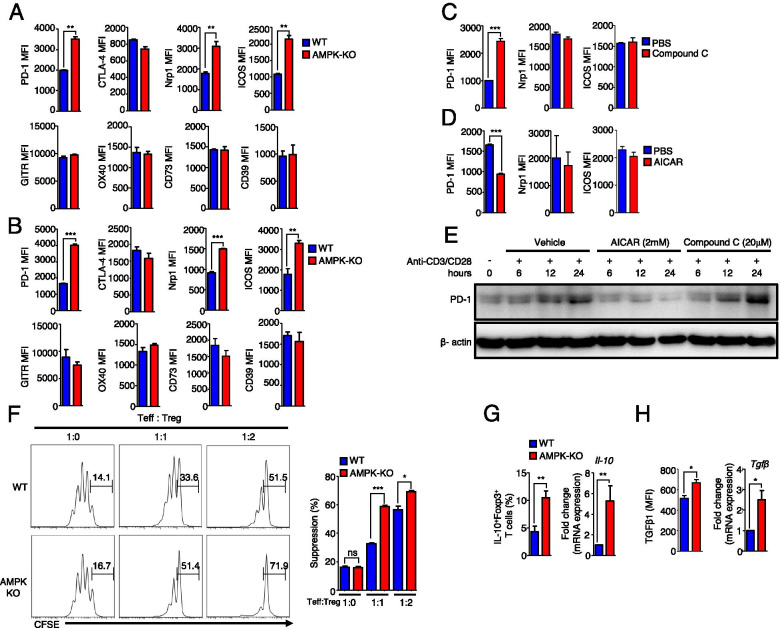
AMPK deficiency in Tregs upregulates the expression of PD-1. Bar diagram representation of flow cytometric analysis of relative mean fluorescence intensities (MFI) of PD-1, CTLA-4, Nrp1, ICOS, GITR, OX40, CD73, and CD39 in Tregs **A** gated from splenocytes of tumor-free mice and **B** gated from tumor-infiltrated lymphocytes. MFI of PD-1, Nrp1, and ICOS in splenic Tregs from WT mice after treatment with **C** compound C or **D** AICAR for 24 h. **E** Immunoblot analysis of PD-1 in Tregs treated with AICAR and compound C at the indicated doses and times of stimulation with anti-CD3/CD28. **F** T cell suppression assays with WT and AMPK-KO Tregs. **G** Bar diagram representation of flow cytometric analysis of IL-10-expressing WT and AMPK-KO Tregs and qRT-PCR results of the *Il-10* mRNA levels. **H** MFI of TGF-β-expressing WT and AMPK- KO Tregs and qRT-PCR results of the *Tgf-β* mRNA levels. The data are presented as the mean ± standard deviation (SD); *n* = 5 mice per group. *P < 0.05; **P < 0.01; ***P < 0.001

Tregs express various inhibitory receptors on their surface [25]. We found that AMPK-KO Tregs expressed higher levels of PD-1, Nrp1, and ICOS than Tregs from WT mice in both tumor-free and tumor-bearing mice (Fig. 2A, B). However, other receptors associated with the suppressive function of Tregs, including CTLA4, GITR, and OX40, were not differentially expressed (Fig. 2A, B). The expression of CD39 and CD73, which contribute to energy depletion in the TME, was also not changed (Fig. 2A, B).

To confirm whether the expression of PD-1, Nrp1, and ICOS in Tregs was regulated by AMPK, we treated WT mice with compound C, a pharmacological inhibitor of AMPK. We found that the expression of PD-1 was significantly upregulated in Tregs after treatment with compound C, whereas no significant alterations were detected in the levels of ICOS and Nrp1 (Fig. 2C). Conversely, treating WT mice with a pharmacological activator of AMPK, AICAR, decreased the expression of PD-1 in Tregs (Fig. 2D). Similar results were obtained with another AMPK activator, metformin (Fig. S6), suggesting that AMPK could restrict the expression of PD-1 in Tregs. Western blot analysis further confirmed that the expression of PD-1 in WT Tregs was reduced after AICAR treatment and increased after compound C treatment in a time-dependent manner (Fig. 2E). We also found that AMPK-KO Tregs inhibited the proliferation of T cells more potently than WT Tregs in a co-culture system (Fig. 2F). Notably, AMPK-KO Tregs expressed higher levels of IL-10 and TGF-β at both the mRNA and protein levels (Fig. 2G, H) than WT Tregs. Collectively, these findings suggest that AMPK suppresses the expression of PD-1 in Tregs, attenuating their immunosuppressive potential.

We also observed that PD-1 expression was higher in CD4^+^ T cells from tumor-bearing mice than tumor-free mice (Fig. S7A). In tumor-free mice, PD-1 expression was comparable between AMPK-deficient CD4^+^ T cells and WT CD4^+^ T cells (Fig. S7B, C). In contrast, in tumor-bearing mice, PD-1 expression was higher in AMPK-deficient CD4^+^ T cells, but not in CD8^+^ T cells (Fig. S7D, E). Therefore, we can presume that AMPK negatively regulates PD-1 expression in CD4^+^ T cells as well as in Tregs.

### Immune checkpoint blockade reduces tumor growth in *AMPK*^fl/fl^*Foxp3*-Cre mice and synergizes with AMPK activation for antitumor immunity

**Fig. 3:**
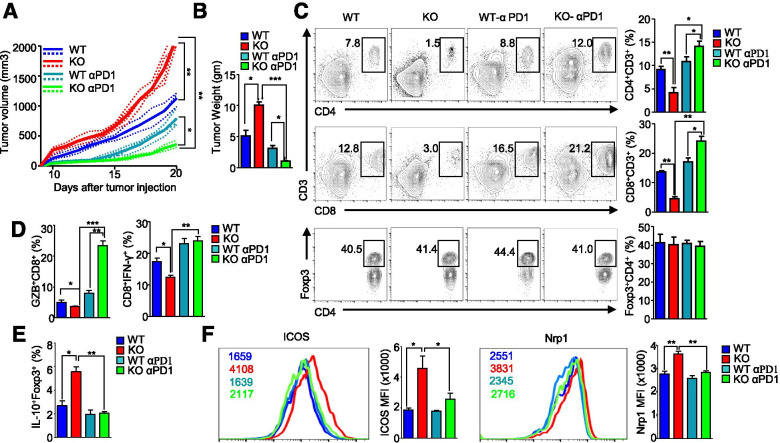
Treatment with anti-PD-1 antibody reduces tumor growth in *AMPK*^fl/fl^*Foxp3*-Cre mice. B16F10 melanoma tumor cells were injected subcutaneously into AMPK-WT and *AMPK*^fl/fl^*Foxp3*-Cre mice. Anti-mouse PD-1 was administered i.p. at 200 μg/mouse at 8, 11, 14, and 17 days after the injection of cancer cells. Tumors were analyzed at day 20 post-tumor transplantation. The **A** volume and **B** weight of the tumors from WT and *AMPK*^fl/fl^*Foxp3*-Cre mice treated with or without anti-PD-1 antibody. **C** Percentage of CD4^+^ and CD8^+^ T cells and CD4^+^Foxp3^+^ Tregs in the tumors of WT and *AMPK*^fl/fl^*Foxp3*-Cre mice treated with or without anti-PD-1 antibody. **D** Flow cytometry analysis of the percentage of GZB- and IFN-γ-producing CD8 ^+^ T cells in lymphocytes isolated from tumor tissues. **E** Bar diagram representation of flow cytometric analysis of the percentage of Foxp3^+^IL-10^+^ T cells. **F** Flow cytometric analysis and MFI of ICOS and Nrp1 in the tumors of WT and *AMPK*^fl/fl^*Foxp3*-Cre mice with or without anti-PD-1 treatment. The data are presented as the mean ± standard deviation (SD); *n* = 5 mice per group. *P < 0.05; **P < 0.01; ***P < 0.001

We reasoned that the increased expression of PD-1 in Tregs might be responsible for the increased tumor growth in *AMPK*^fl/fl^*Foxp3*-Cre mice; therefore, we treated tumor-bearing WT and *AMPK*^fl/fl^*Foxp3*-Cre mice with an anti-PD-1 antibody. Remarkably, the growth of B16F10 tumors in anti-PD-1-treated *AMPK*^fl/fl^*Foxp3*-Cre mice was significantly reduced, compared to that in anti-PD-1-treated WT mice (Fig. 3A, B). Infiltration by CD4^+^ and CD8^+^ T cells was highly increased in *AMPK*^fl/fl^*Foxp3*-Cre mice after anti-PD-1 treatment, whereas no difference was observed for Foxp3^+^ Tregs (Fig. 3C). Similarly, the counts of GZB- and IFN-γ-producing CD8^+^ T cells were highly increased in *AMPK*^fl/fl^*Foxp3*-Cre mice after anti-PD-1 treatment (Fig. 3D). Further, we assessed the expression of IL-10 in Tregs, a hallmark of immunosuppression [26], and found that the counts of IL-10^+^Foxp3^+^ T cells were significantly decreased in *AMPK*^fl/fl^*Foxp3*-Cre mice following anti-PD-1 treatment (Fig. 3E). Notably, anti-PD-1 antibody treatment also significantly limited the expression of ICOS and Nrp1 in AMPK-KO Tregs (Fig. 3F), suggesting that this antibody may work by direct binding to Tregs and by rescuing exhausted T cells. Overall, anti-tumor immunity was more enhanced in *AMPK*^fl/fl^*Foxp3*-Cre mice after anti-PD-1 antibody treatment compared to WT mice, indicating that AMPK is expected to have other functions besides regulating PD-1 expression. When we analyzed Tregs in anti-PD-1 antibody-treated mice, the CD25 expression levels decreased in AMPK-KO Tregs compared to WT Tregs (Fig. S8A). To confirm this, we stimulated anti-CD3/anti-CD28 antibodies-treated Tregs with IL-2 in vitro. Before stimulation, CD25 expression levels were comparable between WT and AMPK-KO Tregs. However, after treatment with anti-PD-1 antibody, CD25 expression levels were slightly reduced in AMPK-KO Tregs (Fig. S8B). Thus, the enhanced anti-tumor immunity in *AMPK*^fl/fl^*Foxp3*-Cre mice after PD-1 treatment may be associated with the regulation of CD25 expression in AMPK-KO Tregs.

Studies have shown that AMPK activators, including AICAR and metformin, can be used to effectively treat several cancers [10, 26]. Given that the activation of AMPK results in the downregulation of PD-1 in Tregs, we next assessed the antitumor effect of combination therapy with AICAR and anti-CTLA4 antibodies. AICAR treatment reduced B16F10 tumor growth in immune-competent mice (Fig. S9A). Additionally, AICAR treatment increased the frequency of CD4^+^ and CD8^+^ T cells in B16F10-bearing C57BL/6 J mice without affecting the percentage of Treg cells (Fig. S9B). The downregulation of PD-1 was also observed in Tregs isolated from tumor tissues following AICAR treatment (Fig. S9C), and combination therapy with AICAR with anti-CTLA4 antibody significantly reduced the tumor growth due to increased antitumor T cell activity in comparison with AICAR monotherapy (Fig. S9A-C). To confirm whether the antitumor effect of AICAR is dependent on T cells, we used immune-deficient *Rag1*^*−/−*^ mice. AICAR had no effect on B16F10 tumor growth in immune-deficient mice (Fig. S9D), which confirmed that the antitumor effect of AICAR was mainly dependent on T cells. Next, we assessed the combination therapy of AICAR with anti-PD-1 antibody. Similar to the treatment with AICAR and anti-CTLA-4 antibody, the combination therapy with AICAR and anti-PD-1 antibody significantly reduced tumor growth due to increased anti-tumor T cell activity in comparison with AICAR monotherapy or anti-PD-1 antibody treatment alone (Figure S10). These findings suggest that the AMPK activation increases the efficacy of anti-CTLA-4 or anti-PD-1 antibody therapy via the potentiation of antitumor immune responses.

### Loss of AMPK in Tregs disrupts metabolic process and enhances the expression of HMGCR

**Fig. 4:**
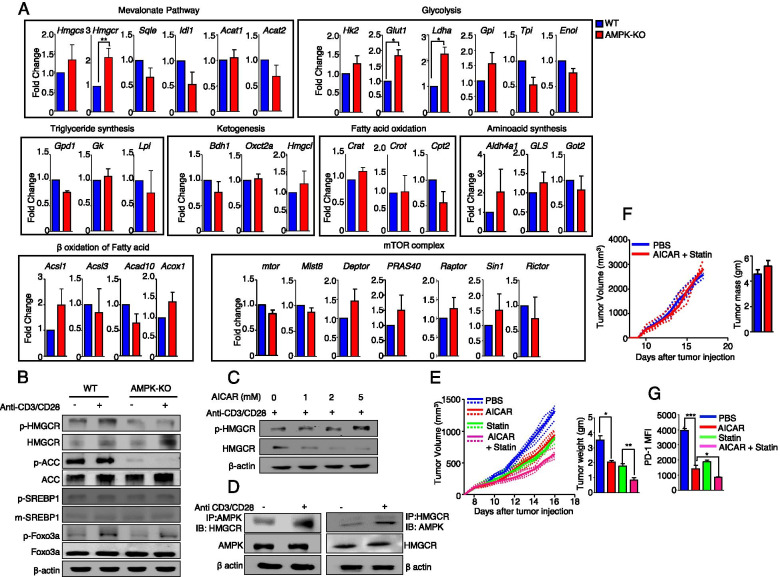
AMPK activation negatively regulates HMGCR in Treg cells. **A** Analysis of the indicated genes in Tregs isolated from WT and *AMPK*^fl/fl^*Foxp3*-Cre mice by RT PCR. **B** Immunoblot analysis of the indicated proteins in WT and AMPK-KO Tregs after 1 h of resting and 4 h of stimulation with anti-CD3/CD28. **C** Detection of the phosphorylated and dephosphorylated form of HMGCR from WT Tregs treated with AICAR at indicated doses after 4 h of stimulation with anti-CD3/CD28. **D** Immunoprecipitation of the lysates of WT Tregs using anti-AMPK and anti-HMGCR antibodies. **E** The volume and weight of B16F10 tumors from WT mice after treatment with PBS, AICAR, statin, and AICAR + statin combination therapy. **F** The volume and weight of B16F10 tumors in *Rag1*^*−/−*^ mice following PBS treatment or co-treatment with AICAR and statin. **G** MFI of PD-1 in Tregs from tumor-bearing WT mice after the indicated treatments. The data are presented as the mean ± standard deviation (SD); *n* = 5 mice per group. *P < 0.05; **P < 0.01; ***P < 0.001

As AMPK is essential for energy homeostasis, AMPK deficiency in Tregs may contribute to the alteration of energy metabolism. We found that the mitochondrial potential and content were significantly reduced in AMPK-KO Tregs (Fig. S11A), suggesting a mitochondrial defect; therefore, we measured the ATP levels in these cells. Although the ATP levels were significantly reduced in AMPK-KO Tregs (Fig. S11B), glycolysis was elevated compared to that in WT Tregs (Fig. S11C). As AMPK was reported to inhibit glycolysis in Tregs by inhibiting mTORC1 signaling [27], we examined CD71 and CD98, key nutrient receptors regulated by mTORC1 [28]. The expression levels of CD71 (*p* = 0.13) and CD98 (*p* = 0.37) were not significantly different between WT and AMPK-KO Tregs (Fig. S11D). Regarding the activation of p70S6 kinase (S6K) and phosphoinositide 3-kinase (PI3K), the phosphorylation levels of S6K and PI3K were markedly increased in WT Tregs, but not in AMPK-KO Tregs after TCR stimulation (Fig. S11E), suggesting that the upregulation of glycolysis in AMPK-deficient Tregs is not mTORC1-mediated. Further, we analyzed the expression of the mTOR complex and of several enzymes associated with energy metabolism. Interestingly, the expression of HMGCR, one of the primary rate-limiting enzymes of the mevalonate pathway, and glycolytic signature genes, including *Glut1* and *Ldha*, was significantly upregulated in AMPK-KO Tregs (Fig. 4A). Western blot analysis revealed that the accumulation of the dephosphorylated form of HMGCR (the active form) was increased in AMPK-KO Tregs, compared to WT Tregs, whereas the expression of phosphorylated-HMGCR (the inactivated form) was decreased, indicating HMGCR activation (Fig. 4B). However, the expression of *ACC, SREBP1*, and *Foxo3a* was not markedly increased in AMPK-KO Tregs compared to WT Tregs after TCR stimulation (Fig. 4B). In addition, the phosphorylated-HMGCR increased, whereas dephosphorylated-HMGCR decreased following AICAR treatment (Fig. 4C), suggesting that HMGCR is a downstream substrate of AMPK in Tregs. Furthermore, we found that AMPK could bind to HMGCR using an immunoprecipitation assay (Fig. 4D). These results suggest that AMPK deficiency induces metabolic changes in Tregs, favoring glycolysis, and that an increase in the HMGCR expression occurred, which may be the underlying reason for the increased tumor growth in *AMPK*^fl/fl^*Foxp3*-Cre mice. Altogether, these findings suggest that AMPK negatively regulates HMGCR activation in Tregs.

Statin, a specific HMGCR inhibitor, was reported to exert antitumor effects in different cancers [29]. Thus, we assessed the antitumor effect of statin in combination with AICAR; WT mice transplanted with B16F10 tumors were treated with AICAR, statin, or both. We observed that the combination therapy led to a reduction in tumor growth, with increased frequencies of CD4^+^ and CD8^+^ T cells and no change in Tregs (Fig. 4E and Fig. S12A). However, reduced tumor growth was not observed in *Rag1*^*−/−*^ mice (Fig. 4F), suggesting that the antitumor effect of this combination therapy depends on T cells and B cells. Indeed, GZB- and IFN-γ-producing CD8^+^ T cells were highly increased after AICAR and statin co-treatment in tumor-bearing C57BL/6 J mice (Fig. S12B). Also, the expression of PD-1 in Tregs was significantly reduced after combination therapy (Fig. 4G). These findings suggest that the activation of AMPK synergizes with the inhibition of HMGCR to suppress tumor growth via the downregulation of PD-1.

### HMGCR regulates the expression of PD-1 via p38 MAPK

**Fig. 5:**
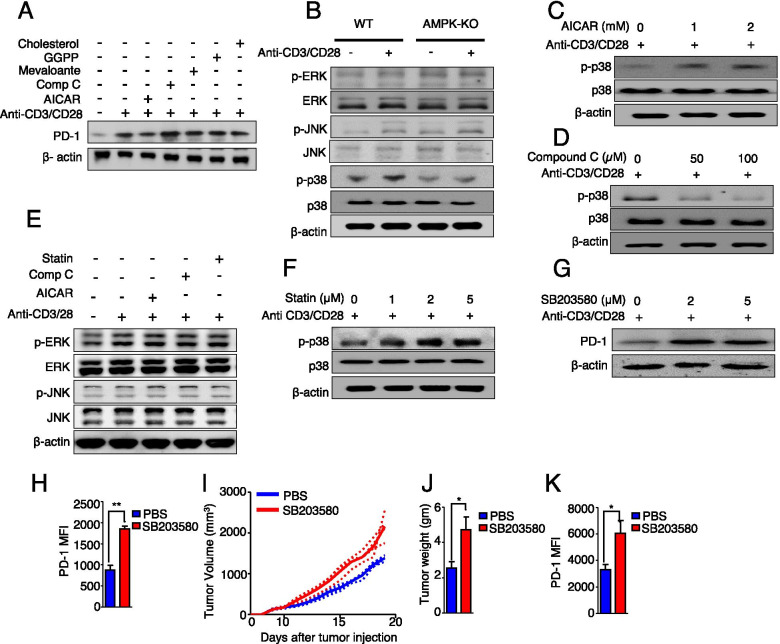
AMPK activates p38 MAPK through the inhibition of HMGCR to regulate the expression of PD-1. **A** Western blot analysis of the expression of PD-1 in treatment with the AMPK modulators: AICAR and compound C and the mevalonate pathway byproducts: cholesterol, GGPP, and mevalonate. **B** Immunoblot analysis of phosphorylated and total ERK, JNK, and p38 MAPK in Tregs from WT and *AMPK*^fl/fl^*Foxp3*-Cre mice after 1 h of resting and 3 h of stimulation with anti-CD3/CD28. Western blot analysis of phosphorylated and total p38 MAPK in WT Tregs treated with or without **C** AICAR or **D** compound C at the indicated doses. **E** Immunoblot analysis of phosphorylated and total ERK and JNK in WT Tregs treated with AICAR, compound C, or statin. **F** Immunoblot analysis of phosphorylated and total form of p38 in Tregs treated with different concentrations of statin. **G** Immunoblot analysis of PD-1 in Tregs treated with SB203580 at the indicated doses. **H** MFI of PD-1 in WT Tregs with or without SB203580 treatment. The **I** tumor volume and **J** weight of B16F10 melanoma tumors in WT mice treated with PBS or SB203580. **K** MFI of PD-1 in Tregs isolated from tumor tissues after treatment with PBS or SB203580. The data are presented as the mean ± standard deviation (SD); *n* = 5 mice per group. *P < 0.05; **P < 0.01; ***P < 0.001

We determined the mechanism underlying HMGCR-mediated PD-1 regulation. We hypothesized that HMGCR might regulate the expression of PD-1 through the mevalonate pathway. Therefore, we assessed the expression of PD-1 in vitro in Tregs treated with the byproducts of the mevalonate pathway, including mevalonate, GGPP, and cholesterol. However, there was no significant difference in the expression of PD-1 after treatment with these byproducts in vitro (Fig. 5A) and in vivo (Fig. S13), suggesting that the HMGCR-mediated regulation of the expression of PD-1 occurs in a mevalonate pathway-independent manner.

Since previous findings suggested that p38 MAPK is involved in the regulation of cholesterol metabolism [30], we investigated whether the canonical MAPK pathway was responsible for the HMGCR-mediated regulation of PD-1. We found no changes in the levels of the phosphorylated and total forms of ERK and JNK, whereas the phosphorylation of p38 was decreased in AMPK-KO Tregs (Fig. 5B). In addition, the level of phosphorylated p38 was increased by treating Tregs with AICAR (Fig. 5C) and decreased by treating with compound C (Fig. 5D), suggesting that AMPK positively regulates p38. Further, the phosphorylation of p38 was significantly increased in Tregs after statin treatment in a dose-dependent manner, whereas no changes were observed in the expression of ERK and JNK (Fig. 5E, F), suggesting that HMGCR negatively regulates the phosphorylation of p38.

We also investigated whether p38 regulates the expression of PD-1. The expression of PD-1 in WT Tregs was significantly increased after treatment with a p38 inhibitor (SB203580) in vitro (Fig. 5G) and in vivo (Fig. 5H). Furthermore, the p38 inhibitor led to increased B16F10 melanoma tumor growth in WT mice in vivo (Fig. 5I, J). Remarkably, the expression of PD-1 in tumor-isolated Tregs was significantly upregulated by SB203580 (Fig. 5K), suggesting that p38 could regulate the expression of PD-1 both in tumor-free and in tumor-bearing mice. Thus, these findings suggest that HMGCR regulates the expression of PD-1 via p38 MAPK.

### Degradation of GSK3β by p38 is involved in the regulation of PD-1

**Fig. 6:**
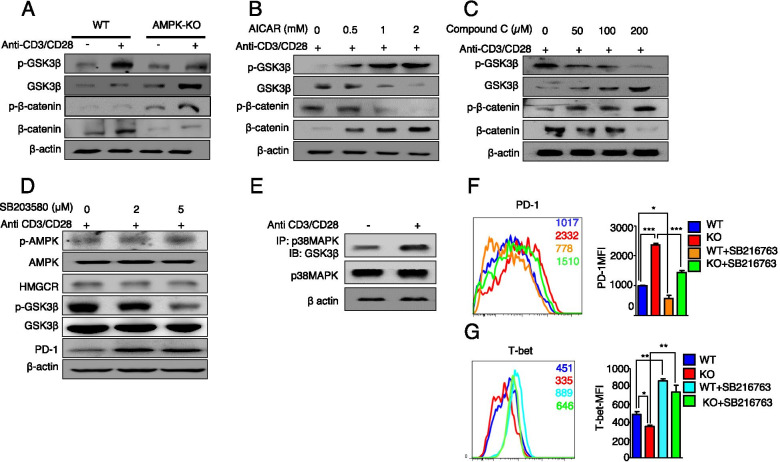
AMPK suppresses the activity of GSK3β through p38 MAPK activation to regulate the expression of PD-1. **A** Analysis of phosphorylated and total GSK3β and β-catenin in WT and AMPK-KO Tregs stimulated with or without anti-CD3/CD28 stimulation for 4 h. Analysis of the expression of GSK3β and β-catenin by western blotting in activated Tregs after **B** AICAR and **C** compound C treatment at the indicated doses. **D** Immunoblot analysis of the indicated proteins in activated Tregs treated with the p38 MAPK inhibitor SB203580 at the indicated doses. **E** Immunoprecipitation assay of the lysates of WT Tregs using anti-p38 and anti-GSK3β antibodies. Flow cytometric analysis of **F** PD-1 and **G** T-bet in Tregs from WT and *AMPK*^fl/fl^*Foxp3*-Cre mice after i.p. treatment with or without the GSK3β inhibitor SB216763 daily for 7 days (2 μg/kg). The data are presented as the mean ± standard deviation (SD); *n* = 5 mice per group. *P < 0.05; **P < 0.01; ***P < 0.001

We identified the likely downstream targets of p38 MAPK regulating the expression of PD-1. Since p38 MAPK regulates the Wnt-β-catenin signaling via the inactivation of GSK3β [31, 32], we first checked the expression of GSK3β and β-catenin in WT and AMPK-KO Tregs. We found that the total form of GSK3β (the active form) was highly increased, whereas phosphorylated GSK3β at Ser9 (the inactivated form) was reduced in AMPK-KO Tregs (Fig. 6A). In accordance, the levels of phosphorylated β-catenin were highly increased, whereas the expression of total β-catenin was decreased in AMPK-KO Tregs, suggesting the degradation of β-catenin and the activation of GSK3β (Fig. 6A). To disclose the potential role of AMPK, we treated WT Tregs with AICAR and compound C. The degradation of GSK3β was enhanced, as evidenced by the increase in the expression of the phosphorylated form after AICAR treatment (Fig. 6B). Conversely, compound C increased the level of total GSK3β and decreased that of phosphorylated GSK3β in Tregs (Fig. 6C). Additionally, the levels of total β-catenin increased after AMPK activation (Fig. 6B) and decreased after AMPK inhibition (Fig. 6C), indicating that AMPK positively regulates the expression of β-catenin and negatively regulates that of GSK3β.

To understand whether p38 MAPK is an upstream regulator of GSK3β, we treated WT Tregs with SB203580. As expected, SB203580 treatment reduced phosphorylated GSK3β, resulting in the accumulation of total GSK3β. Notably, AMPK and HMGCR remained unaffected (Fig. 6D), suggesting that p38 MAPK is an upstream regulator of GSK3β and a downstream effector of AMPK and HMGCR. Furthermore, we observed that p38 MAPK binds to GSK3β (Fig. 6E). Thus, these data suggest that AMPK in Tregs regulates GSK3β via p38 MAPK, thereby inhibiting the expression of PD-1.

To determine whether the increased levels of GSK3β in AMPK-KO Tregs are directly responsible for the upregulation of PD-1 expression, we treated AMPK-KO Tregs with SB216763, a GSK3β inhibitor. Inhibition of GSK3β reduced the expression of PD-1 in WT and AMPK-KO Tregs, compared to their untreated counterparts, albeit AMPK-KO Tregs still exhibit a substantially higher level of PD-1 than WT Tregs, suggesting that AMPK regulates PD-1 expression via both GSK-dependent and -independent mechanisms (Fig. 6F). Previous findings suggested that the inhibition of GSK3β enhanced the expression of T-bet, thereby downregulating the expression of PD-1 in CD8^+^ T cells [33]. Here, we found that T-bet was downregulated in AMPK-KO Tregs (Fig. 6G). Notably, we confirmed this phenotype depended on GSK3β as the expression of T-bet was significantly increased in WT and AMPK-KO Tregs treated with SB216763 (Fig. 6G). Thus, these findings suggest that GSK3β promotes the expression of PD-1 in Tregs through the inhibition of T-bet.

## Discussion

T cell activation is associated with metabolic changes to address the increased energy demand for effector functions. Especially, activated Th1 and Th17 cells mainly depend on glycolysis, controlled by the energy sensor AMPK coordinating energy homeostasis [6, 34]. In cancer cells under metabolic stress, lowered expression of AMPK is associated with increased energy demand, probably due to the low oxygen levels in the TME and the consequent shift toward glycolysis. In this respect, T cells lacking AMPKα1 also display reduced mitochondrial bioenergetics and cellular ATP levels in response to glucose deprivation or to pathogens [2]. Likewise, AMPK is also crucial to maintain the function of Tregs [35]. However, Tregs mainly depend on FAO to survive and function [4, 35]. In this study, we found that the ablation of AMPK in Tregs increased glycolysis and the expression of PD-1, suggesting that AMPK may be modulated in Tregs to promote tumor suppression. Although the regulatory mechanism underlying the expression of PD-1 under conditions of metabolic stress is not fully understood, we suggest that AMPK regulates the expression of PD-1 via the HMGCR/P38 MAPK/GSK3β axis in Tregs.

Tregs are a major barrier to antitumor immunity in various cancers [36]; for instance, they secrete immunoregulatory cytokines, including IL-10 and TGF-β. In addition, Tregs express several inhibitory surface receptors including PD-1, ICOS, and Nrp1 in tumors, mediating immunosuppression [25]. Notably, PD-1 is also expressed in exhausted T cells [37]. In fact, PD-1 blockade is used to treat cancer patients not responding to classical chemotherapy agents, promoting the restoration of the effector functions of exhausted T cells [38, 39]. However, despite the importance of PD-1 in immune regulation, the role of PD-1 in Tregs remains controversial [39, 40]. A recent study suggested that PD-1 expression in Tregs was amplified by PD-1 blockade; consequently, PD-1 blockade accelerated tumor growth [40, 41]. This contradicts our results which shows that the PD-1 blockade in Tregs suppresses tumor growth. However, since antitumor CD4^+^ and CD8^+^ T cells were restored by the PD-1 blockade in our study, it is highly plausible that our phenotype depends mainly on the PD-1 expression in Tregs. In this regard, we presume that the enhanced tumor growth in *AMPK*^fl/fl^*Foxp3*-Cre mice is associated with high levels of PD-1 in Tregs. Notably, the PD-1 blockade exhibited profound anti-tumor effects in murine syngeneic tumor models, particularly in *AMPK*^fl/fl^*Foxp3*-Cre mice. Although anti-PD-1 antibody is expected to act on both Tregs and exhausted T cells, it profoundly exhibited antitumor effects in *AMPK*^fl/fl^*Foxp3*-Cre mice compared to WT mice, suggesting that the increased anti-tumor immunity is due to the PD-1 blockade in Tregs, which is increased in the absence of AMPK signaling.

Despite the importance of PD-1 as a negative feedback regulator of T cell effector functions, the upstream pathway involved in the downregulation of PD-1 is yet unknown. Here, we discovered that AMPK regulates the expression of PD-1 through the HMGCR/p38 MAPK/GSK3β axis. Given that p38 MAPK is regulated by AMPK and by statin, an HMGCR inhibitor [42, 43], we found that HMGCR in Tregs could be regulated by AMPK. Consistently, we found that the inhibition of HMGCR by statin activates p38 and suppresses the expression of PD-1 in Tregs, suggesting that HMGCR is an upstream regulator of p38MAPK. However, the detailed mechanism underlying the HMGCR-mediated regulation of p38 MAPK needs to be further explored. Additionally, previous studies suggested that p38 MAPK inactivates GSK3β via phosphorylation [32]. We also found that p38 MAPK interacted with GSK3β and promoted its phosphorylation, suppressing the expression of PD-1; these data suggest that GSK3β is a downstream substrate of p38 in Tregs. Interestingly, our data suggest that GSK3β upregulates the expression of PD-1 in Tregs via the inhibition of T-bet, in line with data reported in CD8^+^ T cells [36]. Notably, GSK3β-mediated phosphorylation enhanced the proteasomal degradation of β-catenin, a key mediator of Wnt signaling [44]. Activated Tregs increased the expression of β–catenin, but it was attenuated in LKB1-deficient Tregs [45]. In this regard, the decreased levels of β-catenin detected in AMPK-KO Tregs may explain the increased expression of PD-1; However, it is still unclear how GSK3β regulates PD-1 expression directly. One hypothesis is that GSK3β-mediated ER stress can induce PD-1 expression. GSK-3β inhibition increases IRE1α-dependent XBP1 splicing, which directs the transcription of several genes involved in the functional and structural expansion of the endoplasmic reticulum (ER) and genes associated with the ER-associated degradation (ERAD) pathway in order to reduce ER stress and restore ER homeostasis [46]. In addition, another study reported that activated XBP1 binds to the Pdcd1 promoter and 2B4 promoter to regulate the expression of inhibitory receptors in CD8^+^ T cells [47] however, the level of PD-1 expression was not changed in XBP1-deficient CD4^+^ T cells [48]. Our preliminary data suggested that the expression of the spliced form of XBP1 was increased in AMPK-deficient CD4 T cells (data not shown). However, the mechanism of XBP1-mediated PD-1 expression is still unclear and further studies are needed to elucidate the mechanism.

LKB1 is a master kinase that functions upstream of AMPK, along with TAK1 and CaMKKβ, which acts as a potent tumor suppressor that directly phosphorylates and activates AMPK [8]. However, our previous study suggested that the phenotype of Treg-specific *AMPK*^fl/fl^*Foxp3*-Cre mice differs from Treg-specific LKB1-KO mice, as only Treg-specific LKB1-KO mice developed spontaneous autoimmune inflammation [49]. Interestingly, LKB1-KO Tregs showed hyperactivation of mTORC1 signaling [45], while AMPK-KO Tregs did not. Moreover, the loss of LKB1 in Tregs reduced *HMGCR* expression [45], but the loss of AMPK in Tregs increased *HMGCR* expression. Thus, we presumed that the role of AMPK is distinct from that of LKB1 in CD4^+^ T cells. Additionally, AMPK expression was significantly reduced in tumor-infiltrating Tregs of WT mice, whereas there was no significant change in LKB1 levels. This finding suggests that AMPK exerts a tumor-suppressive effect independent of LKB1 in Tregs. Collectively, it can be presumed that AMPK attenuates the immune suppressive function of Tregs in the TME, thereby potentiating antitumor immunity.”

Inhibitory immune checkpoint blockade using anti-PD-1 and anti-CTLA4 antibodies has provided substantial benefits to certain cancer patients [50]. However, the monotherapy clinical outcomes are not satisfactory yet. Several approaches are therefore being considered to improve the efficacy of these immune checkpoint inhibitors. For instance, combined therapy with existing anticancer therapies, such as chemotherapy, radiotherapy, and targeted therapy, was proposed [51]. However, such combined therapy could induce severe toxicity and side effects. Our findings showed that the AMPK activator, AICAR, exhibited synergistic antitumor effects when combined with anti-PD-1 and anti-CTLA4 antibodies, suggesting that AMPK activators could be considered to complement anti-PD-1 and anti-CTLA4 therapy to improve the outcomes of cancer patients. In addition, HMGCR inhibitors have been widely used as cholesterol-targeting drugs in clinical studies in cancer patients [29]. In our study, a synergistic effect was observed when statin was combined with AICAR, suggesting that AMPK activation combined with the inhibition of HMGCR could be a potential combination therapy for cancer treatment.

## Conclusion

In conclusion, we demonstrated the role of AMPK in Tregs in regulating antitumor immunity: AMPK promotes antitumor immunity due to the downregulated expression of PD-1 via the HMGCR/P38 MAPK/GSK3β axis. The activation of AMPK combined with anti-PD-1 and anti-CTLA4 antibodies or with a HMGCR inhibitor exhibited synergic anti-cancer activity in murine tumor models, supporting their potential clinical use. Altogether, our findings provide support for the notion that AMPK in Tregs acts not only as a crucial regulator maintaining metabolic homeostasis, but also as a potent tumor suppressor.

## Supplementary Information

**Additional file 1: Table 1.** The real-time PCR primer list, along with the sequences.

**Additional file 2: Figure S1.** The correlation between AMPK and PD-1 mRNA expression in multiple cancer patient datasets. **Figure S2.** The phenotype of *AMPK*^fl/fl^*Foxp3*-Cre mice is similar with that of WT mice. **Figure S3.** Loss of AMPK in Tregs promotes TC-1 and MC38 tumor growth. **Figure S4.** AMPK deficiency in Tregs reduces antitumor T cell populations. **Figure S5.***AMPK*^fl/fl^*Foxp3*-Cre mice show low GZB^+^ CD8 T cell numbers per unit area. **Figure S6.** Metformin inhibits the expression of PD-1. **Figure S7.** Loss of AMPK enhances the expression of PD-1 in tumor infiltrated CD4^+^ T cells. **Figure S8.** Deficiency of AMPK causes down-regulation of CD25 expression by anti-PD1 antibody treatment. **Figure S9.** AMPK activation synergizes with CTLA4 checkpoint blockade to limit B16F10 tumor growth. **Figure S10.** AMPK activation synergizes with PD-1 checkpoint blockade to suppress tumor growth. **Figure S11.** Deficiency of AMPK in Tregs changes metabolic process with independent of mTORC1 signaling. **Figure S12.** Combined therapy of AICAR and Statin increases effector T cells. **Figure S13.** PD-1 expression in splenic Tregs from WT mice treated with mevalonate pathway by products in vivo.

## Data Availability

Further information and requests for reagents and resources should be directed to and will be made available by the lead contact Jae-Hoon Chang (jchang@yu.ac.kr) upon reasonable request. The data supporting the conclusions of this article have been provided in this article and its supplementary files.

## Abbreviations

ACC: Acetyl-CoA carboxylase
ADP: Adenosine diphosphate
AICAR: 5-Aminoimidazole-4-carboxamide riboside
AMPK: Adenosine monophosphate-activated protein kinase
ATP: Adenosine triphosphate
CFSE: Carboxyfluorescein diacetate succinimidyl ester
CTLA4: Cytotoxic T-lymphocyte associated protein 4
CTLs: Cytotoxic T lymphocytes
CTV: Cell trace violet
dLN: Draining lymph node
FAO: Fatty acid oxidation
FOXO: Forkhead box O
Foxp3: Forkhead box P3
GGPP: Geranylgeranyl pyrophosphate
GITR: Glucocorticoid induced tumor necrosis factor receptor
Glut1: Glucose transporter type 1
GSK3β: Glycogen synthase kinase 3 beta
GZB: Granzyme B
HMGCR: 3-hydroxy-3-methylglutaryl-CoA reductase
IACUC: Institutional Animal Care and Use Committee
ICOS: Inducible T-cell Costimulator
IFN-γ: Interferon gamma
IL: Interleukin
i.p.: Intraperitoneally
LKB1: Liver kinase B1
MAPK: Mitogen activated protein kinase
mTORC1: Mammalian target of rapamycin complex 1
Nrp1: Neuropilin1
PD-1: Programmed cell death protein-1
PD-L1: Programmed death ligand-1
RNA: Ribonucleic acid
SREBP: Sterol regulatory element binding protein
TGF-β: Transforming growth factor beta
Th: T-helper cells
TIM-3: T cell immunoglobulin and mucin domain containing
TME: Tumor microenvironment
Tregs: Regulatory T cells
