# Supplementary material for: AMPK promotes antitumor immunity by downregulating PD-1 in regulatory T cells via the HMGCR/p38 signaling pathway
Source: Mol Cancer. 2021 Oct 14;20:133. doi: 10.1186/s12943-021-01420-9 (PMC8515644; doi:10.1186/s12943-021-01420-9)
Supplement: Supplementary file 1 — Additional file 1: Table 1. The real-time PCR primer list, along with the sequences. [file 12943_2021_1420_MOESM1_ESM.docx]

| **Table 1**: The real-time PCR primer list, along with the sequences | | | |
| --- | --- | --- | --- |
| **Species** | **Genes** | **Forward (5**'**-3**'**)** | **Reverse (5**'**-3**'**)** |
| mouse | *Hmgcr* | TGCTGCTTT GGCTGTATGTC | TGAGCGTGAACAAGAAGAACCAG |
| mouse | *Hmgcs* | GTCCCTCCACAAATGACCAC | ATGACAGCCGACTCAGGTTC |
| mouse | *Hmgcl* | ACTACCCAGTCCTGACTCCAA | TAGAGCAGTTCGCGTTCTTCC |
| mouse | *Sqle* | TGGTGGAGGAATGACAGTCG | AAGCAAGCTTTTCGGAGCTG |
| mouse | *Idl1* | CACTGGCAGGAGTGATTGGA | TTGCTGGCATTGATTTCAGG |
| mouse | *Bdh1* | AAGCACTGGAAGCAGACACAT | ACACTTAGGGCTTTTCCTGGG |
| mouse | *Oxct2a* | GTGGACGTGGGTACTTTCG | ACCATCTAGCAGAAGGAAGCTG |
| mouse | *Gpd1* | GTTGCCTAATCCAGACGTGTG | AGATAGCTCTGACGTGTG |
| mouse | *Acat1* | GAAACCGGCTGTCAAAATCTGG | TGTGACCATTTCTGTATGTGTCC |
| mouse | *Acat2* | ACAAGACAGACCTCTTCCCTC | ATGGTTCGGAAATGTTCACC |
| mouse | *Crat* | TGCTGCCAGAACCGTGGT | TCCAGGGATTGCTGAAGTGG |
| mouse | *Crot* | CGAACAGAGACTGTGCGATCTT | CATCTTTTGCTGACGTTCAAGG |
| mouse | *GK* | ACCGCAAGCAGATCTACAAC | TGGGGTGCAGCTTGTACA |
| mouse | *Lpl* | GTGACCAAGGTAGACCAGCC | GAAGAGACTTCAGGCAGCTG |
| mouse | *Got2* | GTTGAAATGGGACCTCCAGA | GGGCAGGTATTCTTTGTCCA |
| mouse | *Hk2* | ATTGTCCAGTGCATCGCGGA | AGGTCAAACTCCTCTCGCCG |
| mouse | *Glut1* | TCAACACGGCCTTCACTG | CACGATGCTCAGATAGGACATC |
| mouse | *Ldha* | ACAGTTGTTGGGGTTGGTGC | CGCAGTTACACAGTAGTCTTTG |
| mouse | *Gpi* | GTCCCCGGGTCTGGTTTG | GGTGACCTTTTCAGTCTTCG |
| mouse | *Eno1* | AGATCCCTTTGACCAGGACGA | AGCCTTGG CAATCCGCTTA |
| mouse | *Cpt2* | CAGTGCACAGAAGCCTCTCTTG | CTTCCCAATGCCGTTCTCAA |
| mouse | *Aldh4a1* | CATAATCCAGTTTGTGCCAG | TCCACAGGTGTTTGAAGGTG |
| mouse | *GLS* | TTCGCCCTCGGAGATCCTAC | CCAAGCTAGGTAACAGACCCT |
| mouse | *Acsl1* | ATCTGGTGGAACGAGGCAAG | TCCTTTGGGGTTGCCTGTAG |
| mouse | *Acsl3* | TGTCTTTCTCATGGATGCCGA | CAGCACGGATGTGTCTCCTT |
| mouse | *Acad10* | GCCTGGCCGGATTCATC | GGACATCACCCGTGTCTTCAT |
| mouse | *Acox1* | AGCCTCTGCCAGGCATCAC | CATCAACATGTTCTCTCTAG |
| mouse | *mTOR* | TCCTGCGCAAGATGCT CATC | TGTGCTCCAGCTCTGTCAGGA |
| mouse | *Mlst8* | AGCGTGTGTTAAGTGCAGGT | AGTCCTGATGCTGCACTGTT |
| mouse | *Deptor* | AGCAGAGAGAGCTGGAACGC | CAGAGGCCTCCTTATGTTCA |
| mouse | *PRAS40* | TCAATACCAGCGACTTCCAGA | TGACCCTTGGAGCGTTTAGA |
| mouse | *Raptor* | AGATTGTGAAGGGGCTGACA | ACGCTTCTCCACCGAATACA |
| mouse | *Rictor* | ACTTGTCCTCTGTCGCTTCA | AGCCTCACTTCATGCTTCTT |
| mouse | *Sin1* | TCGATTGTGACCTGCTCTGT | AAGCTTGTTCGCCTGTTCAG |
| mouse | *IL-10* | ATAACTGCACCCACTTCCCAGTC | CCCAAGTAACCCTTAAAGTCCTGC |
| mouse | *TGFβ1* | CATTCCAGCTGTACACTGCAG | AGGGGTGGAGATGTAGTTTGG |
| mouse | *Prkaa1* | GCTGTGGCTCACCCAATTAT | TGTTGTACAGGCAGCTGAGG |
