# Supplementary material for: AMPK promotes antitumor immunity by downregulating PD-1 in regulatory T cells via the HMGCR/p38 signaling pathway
Source: Mol Cancer. 2021 Oct 14;20:133. doi: 10.1186/s12943-021-01420-9 (PMC8515644; doi:10.1186/s12943-021-01420-9)
Supplement: Supplementary file 2 — Additional file 2: Figure S1. The correlation between AMPK and PD-1 mRNA expression in multiple cancer patient datasets. Figure S2. The phenotype of AMPKfl/flFoxp3-Cre mice is similar with that of WT mice. Figure S3. Loss of AMPK in Tregs promotes TC-1 and MC38 tumor growth. Figure S4. AMPK deficiency in Tregs reduces antitumor T cell populations. Figure S5.AMPKfl/flFoxp3-Cre mice show low GZB+ CD8 T cell numbers per unit area. Figure S6. Metformin inhibits the expression of PD-1. Figure S7. Loss of AMPK enhances the expression of PD-1 in tumor infiltrated CD4+ T cells. Figure S8. Deficiency of AMPK causes down-regulation of CD25 expression by anti-PD1 antibody treatment. Figure S9. AMPK activation synergizes with CTLA4 checkpoint blockade to limit B16F10 tumor growth. Figure S10. AMPK activation synergizes with PD-1 checkpoint blockade to suppress tumor growth. Figure S11. Deficiency of AMPK in Tregs changes metabolic process with independent of mTORC1 signaling. Figure S12. Combined therapy of AICAR and Statin increases effector T cells. Figure S13. PD-1 expression in splenic Tregs from WT mice treated with mevalonate pathway by products in vivo. [file 12943_2021_1420_MOESM2_ESM.pdf]

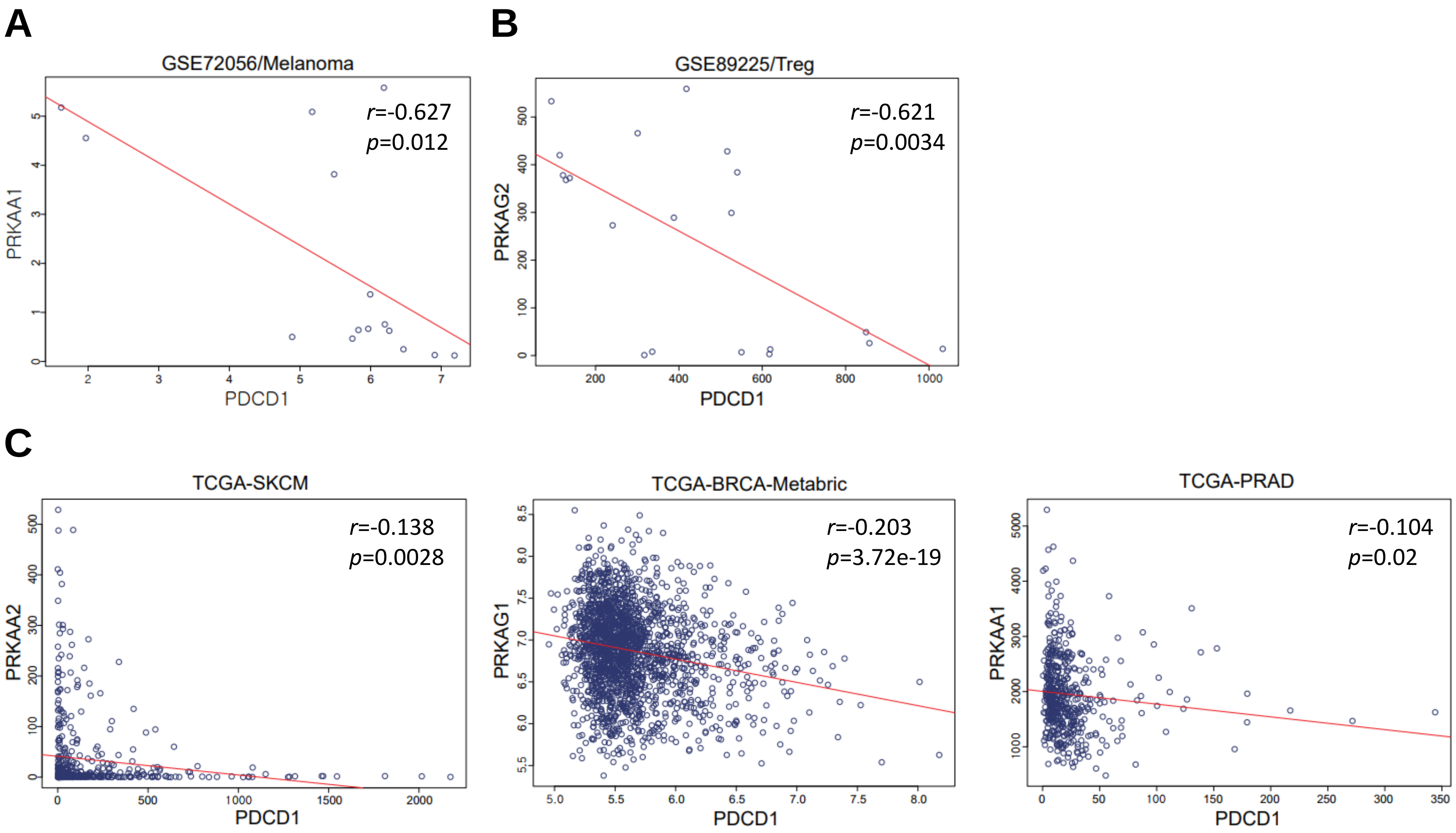

Figure S1. The correlation between AMPK and PD-1 mRNA expression in multiple cancer patient datasets. (A) In single-cell RNA-seq dataset which was assessed from melanoma tissues (GSE72056), we downloaded the TPM dataset file and sorted any values more than 3 TPM for cells that highly expressed Foxp3 transcripts and excluded invalid TPM values for *Pdcd1* and *Prkaa1* expression (r = -0.627, p=0.012). (B) Gene expression profiles of *Prkag2* and *Pdcd1* were analyzed in resident Tregs in human breast cancer specimens using the available datasets (GSE89225) (r=-0.621, p=0.0034). (C) Normalized counts of AMPK subunit genes and *Pdcd1* in tumors was analyzed in TCGA database obtained form melanoma (TCGA-SKCM; skin cutaneous melanoma, r=-0.138, p=0.0028), breast cancer (TCGA-BRCA-Metabric; breast invasive carcinoma, r=-0.203, p=3.73e-19), prostate cancer (TGCA-PRAD; prostate adenocarcinoma, r=-0.104, p=0.02) patients.

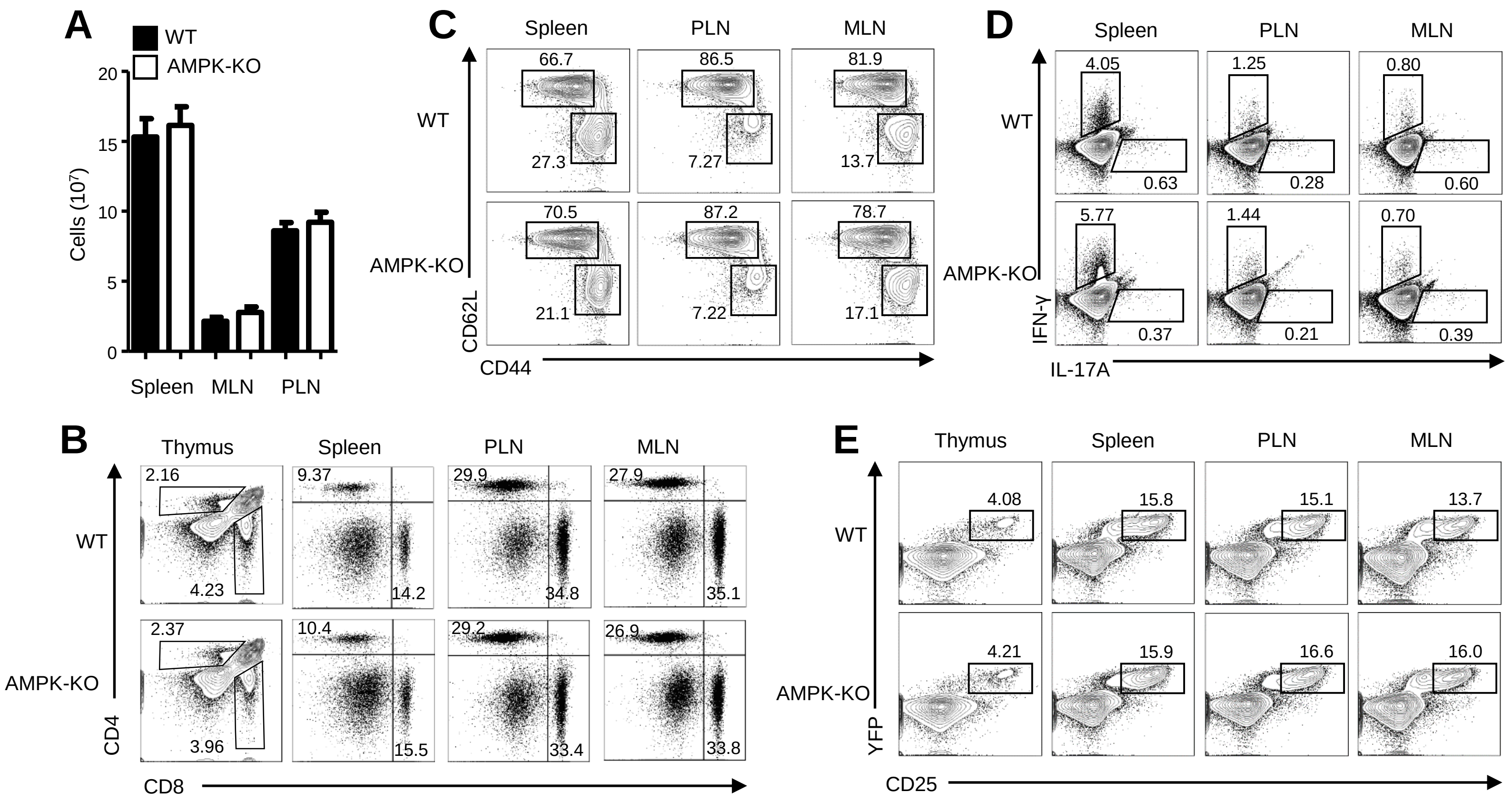

Figure S2. The phenotype of *AMPK*$^{fl/fl}$*Foxp3*-Cre mice is similar with that of WT mice. (A) Total cell number in spleen, MLN, and PLN of WT and AMPK-KO mice. **(B)** Percentage of $CD4^+$ and $CD8^+$ T cells in thymus, spleen, PLN, and MLN of WT and AMPK-KO mice. **(C)** Expression of CD62L and CD44 in $CD4^+$ T cells from spleen, PLN, and MLN of WT and AMPK-KO mice. **(D)** Percentage of IFN-γ- and IL-17A-producing $CD4^+$ T cells in lymphocytes isolated from spleen, PLN, and MLN. **(E)** Percentage of YFP^+CD25^+ Tregs in thymus, spleen, PLN, and MLN from WT and AMPK-KO mice.

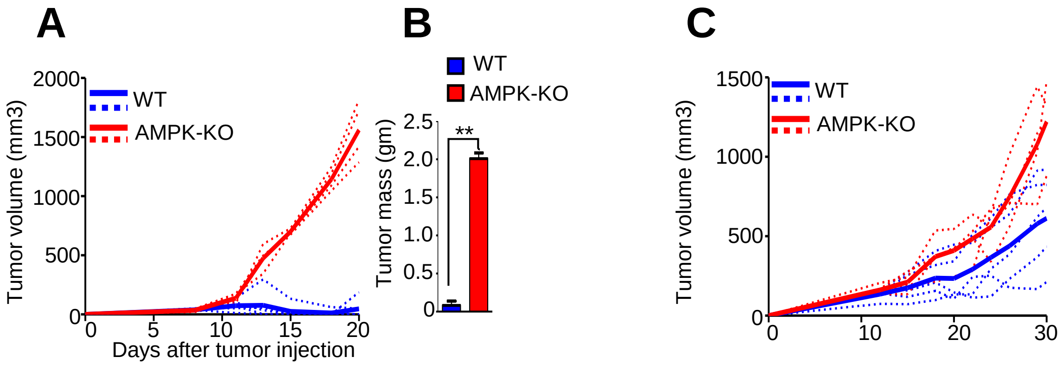

Figure S3. Loss of AMPK in Tregs promotes TC-1 and MC38 tumor growth.
(A) Tumor volume and **(B)** tumor weight of the WT and *AMPK*$^{fl/fl}$*Foxp3*-Cre mice after s.c. inoculation with TC-1 cervical cancer cells. **(C)** Tumor volume of WT and *AMPK*$^{fl/fl}$*Foxp3*-Cre mice which were s.c. injected with MC38 colon adenocarcinoma cells. The data are presented as the mean ± standard deviation (SD). $^{**}P < 0.01$.

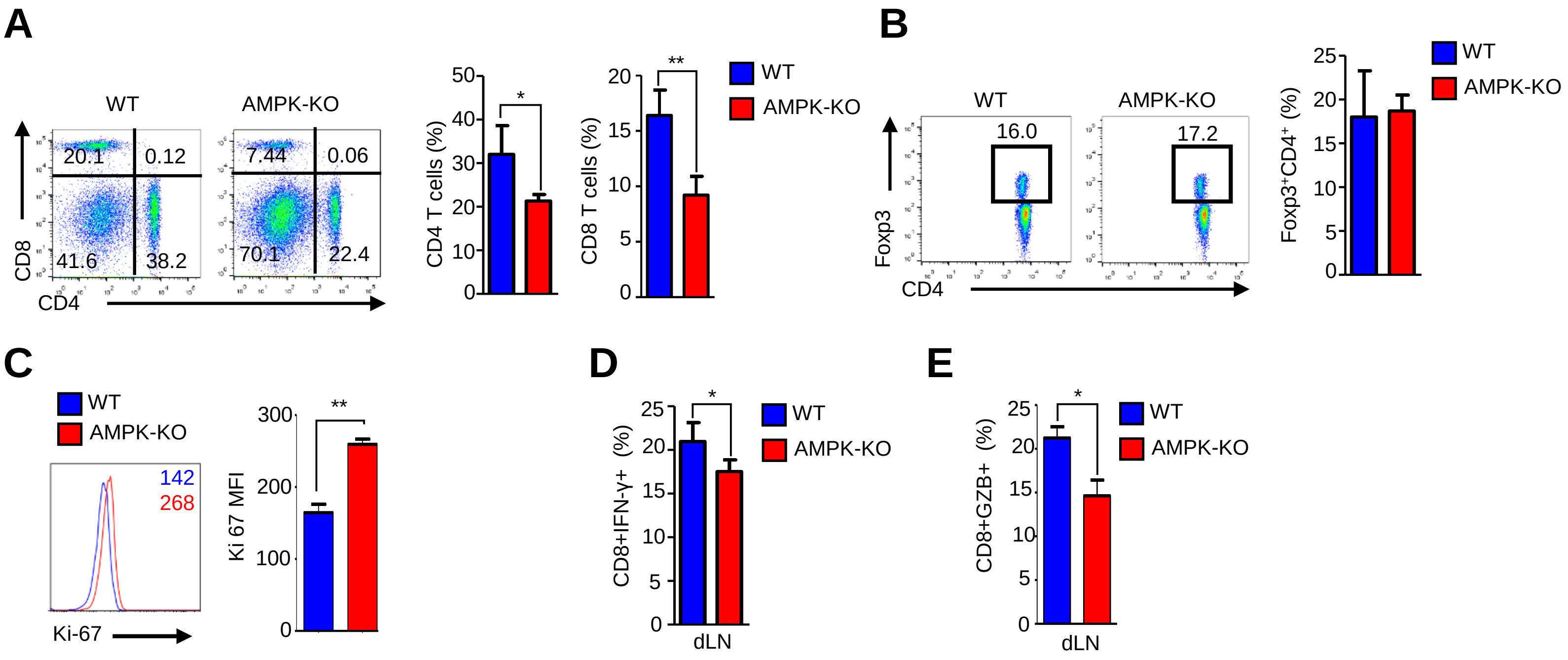

Figure S4. AMPK deficiency in Tregs reduces antitumor T cell populations. (A) Flow cytometric analysis of CD4^{+} and CD8^{+} T cells and **(B)** of CD4^{+}Foxp3^{+} Tregs from the draining lymph nodes of WT and *AMPK*$^{fl/fl}$*Foxp3*-Cre mice injected with B16F10 melanoma cells. (**C**) Ki-67 protein expression in Tregs from WT and *AMPK*$^{fl/fl}$*Foxp3*-Cre mice. Flow cytometric analysis of the percentage of **(D)** IFN-γ- and **(E)** GZB-producing CD8^{+} T cells in the draining lymph nodes of tumor bearing WT and *AMPK*$^{fl/fl}$*Foxp3*-Cre mice. The data are presented as the mean ± standard deviation (SD). *$P < 0.05$; **$P < 0.01$.

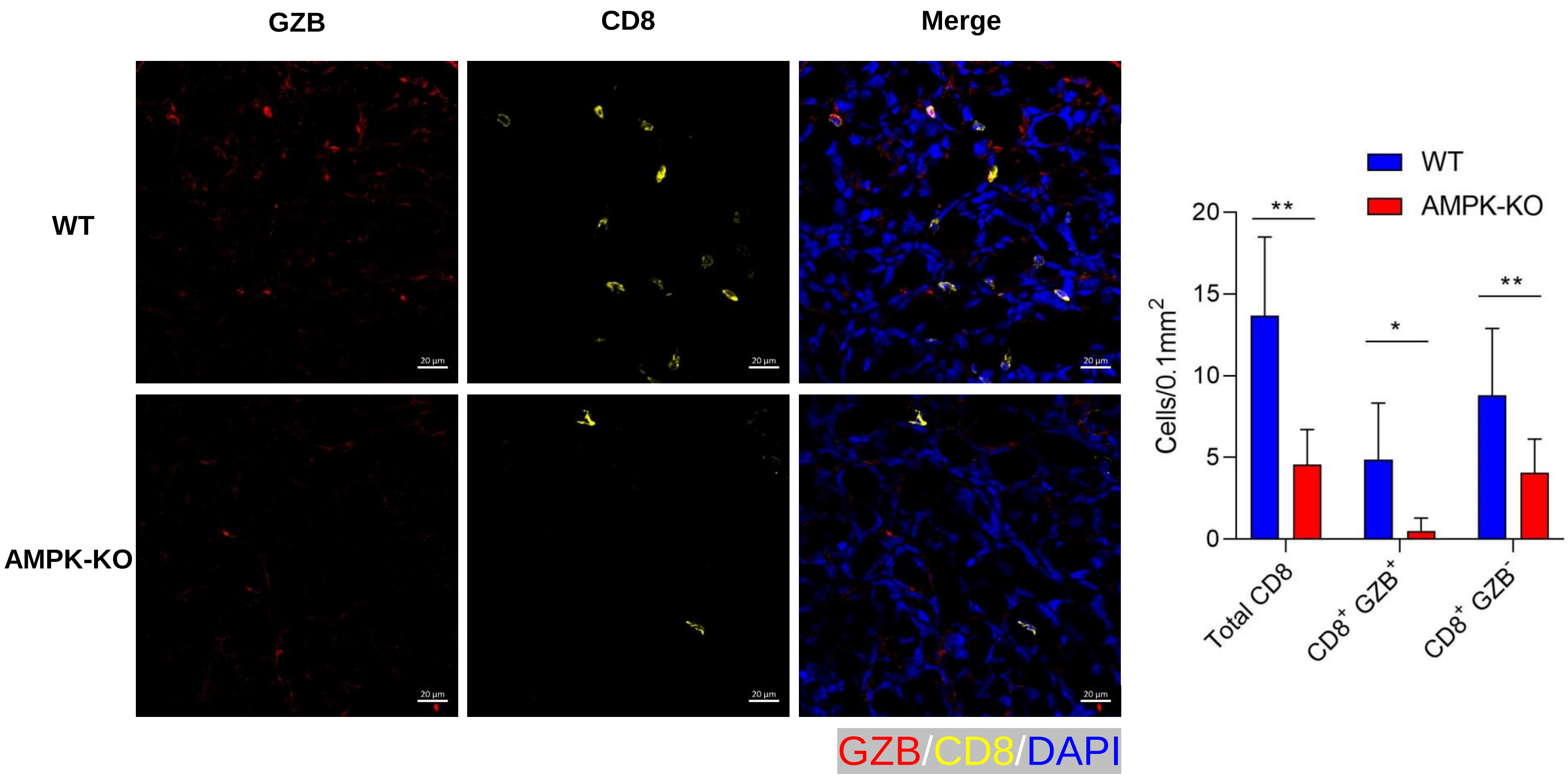

Figure S5. ***AMPK*[fl/fl]*Foxp3*-Cre mice show low GZB[+] CD8 T cell numbers per unit area.** Analysis of GZB[+] CD8[+] T cells numbers per unit area in stained TC-1 tumor tissue excised from WT and AMPK-KO mice by confocal microscopy. The data are presented as the mean ± standard deviation (SD). $^{**}P < 0.01$; $^{***}P < 0.001$.

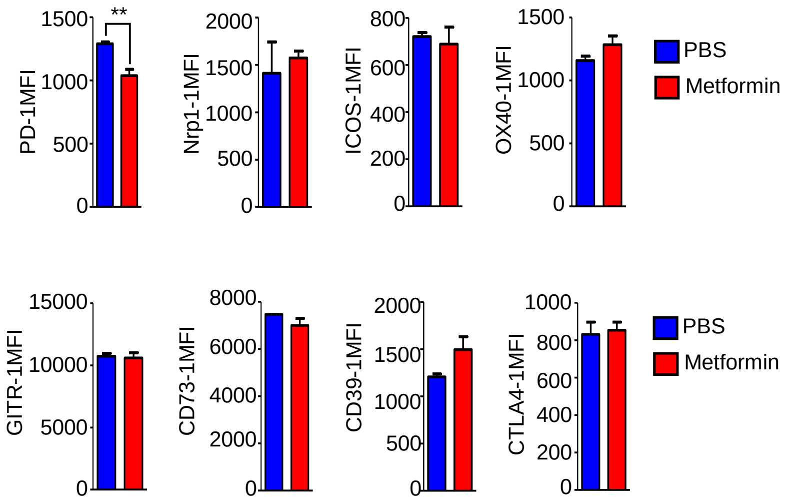

Figure S6. Metformin inhibits the expression of PD-1. Flow cytometric analysis and MFI of the indicated markers in splenic Tregs after i.p injection with 50 mg/kg of metformin for 24 hours in C57BL/6. The data are presented as the mean ± standard deviation (SD). $^{**}P < 0.01$.

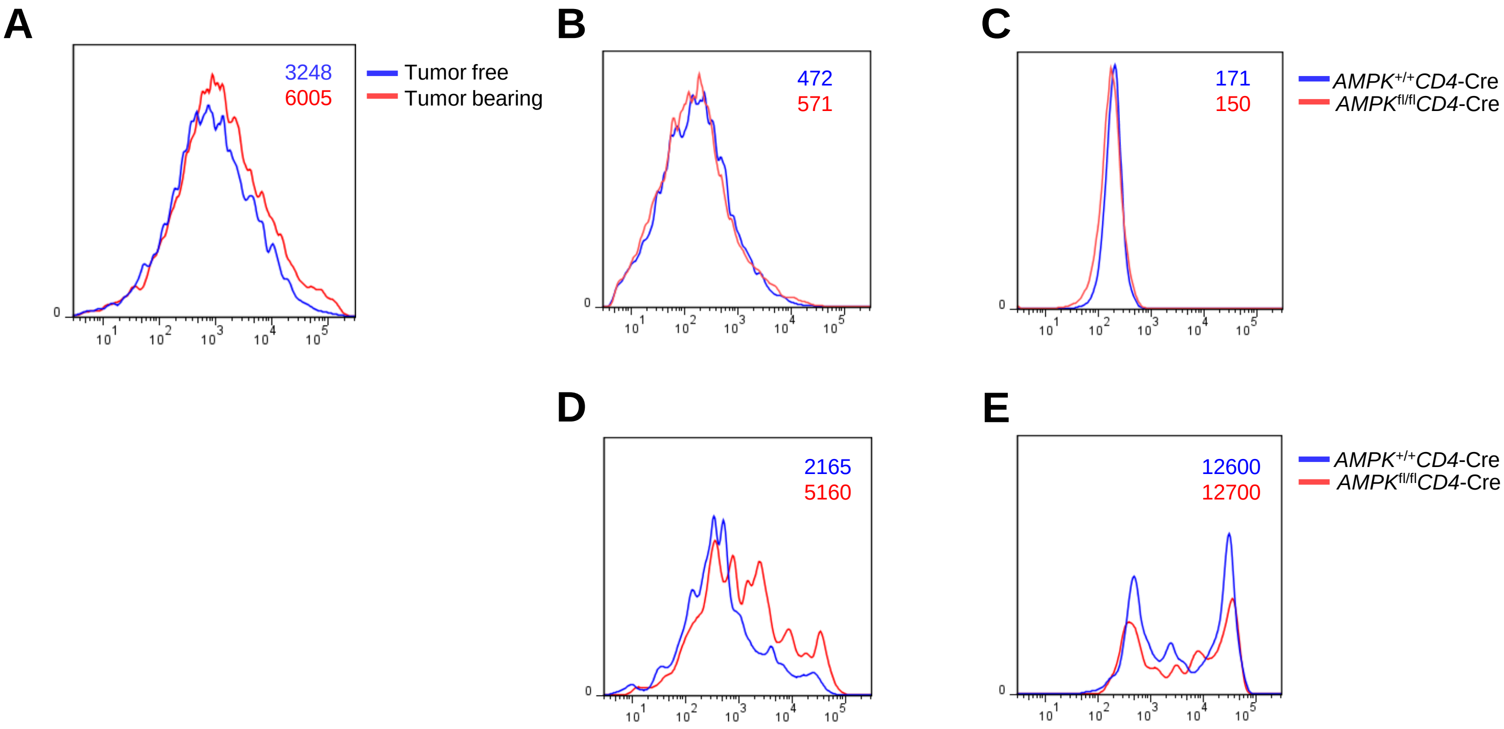

Figure S7. Loss of AMPK enhances the expression of PD-1 in tumor infiltrated CD4$^+$ T cells.

(A) Flow cytometry analysis of PD-1 expression on CD4$^+$ T cells from tumor-free and tumor-bearing mice. Flow cytometry analysis of PD-1 expression in splenic **(B)** CD4$^+$ and **(C)** CD8$^+$ T cells from WT and *AMPK*$^{fl/fl}$*CD4*-Cre mice. Flow cytometry analysis of PD-1 expression in **(D)** CD4$^+$ and **(E)** CD8$^+$ T cells from tumor tissues of WT and AMPK-KO mice.

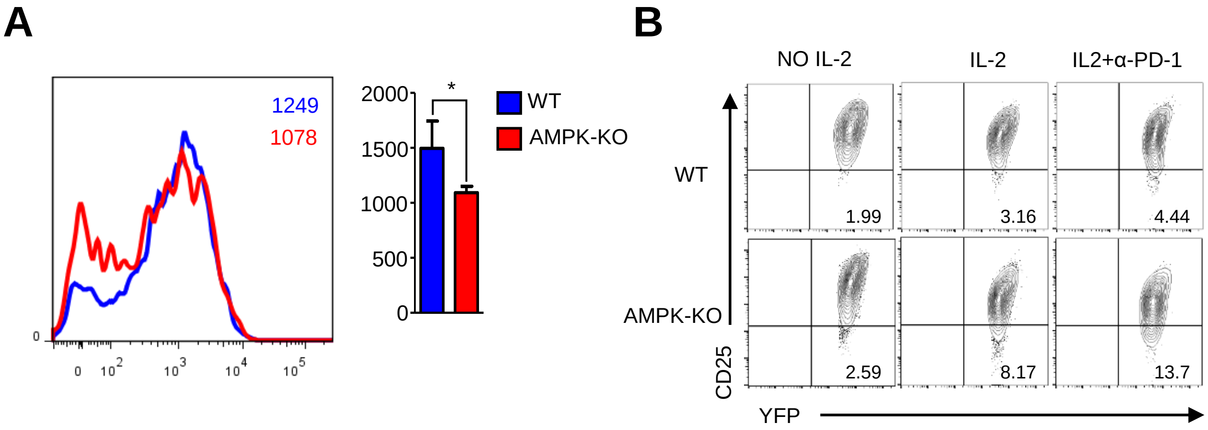

Figure S8. Deficiency of AMPK causes down-regulation of CD25 expression by anti-PD1 antibody treatment. (A) Histogram and MFI of CD25 expression on Tregs of WT and AMPK-KO mice after anti-PD-1 antibody treatment. (B) Percentage of $CD25^{low}$ cells among YFP^{+} Tregs which were sorted from WT and AMPK-KO mice and stimulated with IL-2 alone or IL-2 plus anti-PD-1 antibody.

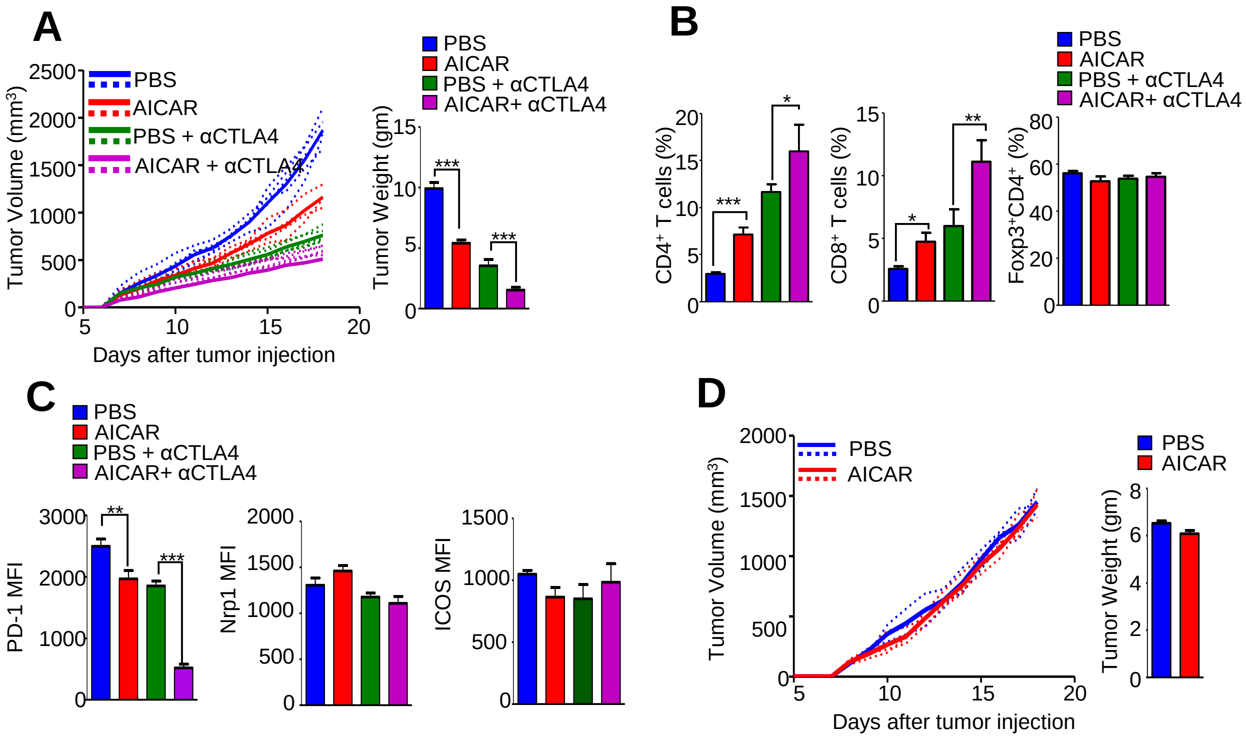

Figure S9. AMPK activation synergizes with CTLA4 checkpoint blockade to limit B16F10 tumor growth. (A) The volume and weight of tumors formed by B16F10 melanoma cells in C57BL/6J mice treated with PBS, AICAR at 500 mg/kg/day, anti-CTLA4 antibody at 100 μg/mouse or the combination, n=5 mice per group. **(B)** Percentage of $CD4^+$ and $CD8^+$ T cells and $CD4^+Foxp3^+$ Tregs in lymphocytes isolated from tumors collected from C57BL/6J mice after treatment with PBS, AICAR, anti-CTLA4 antibody, or the combination. **(C)** Flow cytometric analysis and MFIs of PD-1, ICOS, and Nrp1 in Tregs isolated from B16F10 tumor tissues. **(D)** Tumor growth and tumor weight formed by B16F10 melanoma cells in B16F10 *Rag1*$^{-/-}$ mice after treatment with PBS or AICAR. The data are presented as the mean ± standard deviation (SD); n= 5 mice per group. *P < 0.05; **P< 0.01.

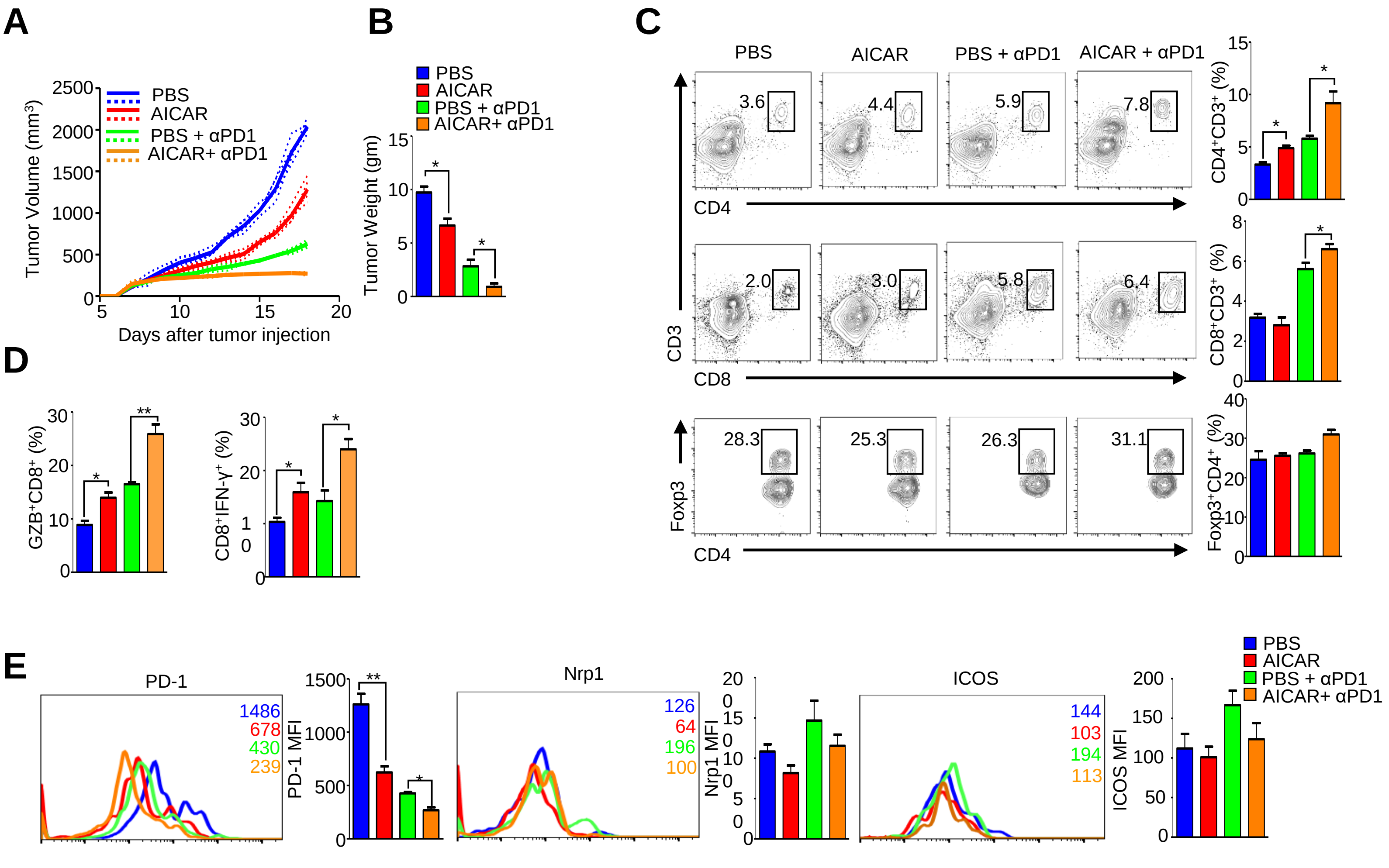

Figure S10. AMPK activation synergizes with PD-1 checkpoint blockade to suppress tumor growth. (A) Tumor volume and (B) Tumor volume of B16F10 melanoma in C57BL/6J mice treated with PBS, AICAR, anti-PD-1 or the combination, n=5 mice per group. (C) Percentage of $CD4^+$, $CD8^+$ T cells and $CD4^+Foxp3^+$ Tregs in lymphocytes isolated from B16F10 tumors of C57BL/6J mice treated with AICAR, anti-PD-1 or combination. (D) Analysis of percentage of GZB- and IFN-γ-producing $CD8^+$ T cells in lymphocytes isolated from tumors by flow cytometry. Histogram and MFI of (E) PD-1, (H) Nrp1, and (I) ICOS expression in lymphocytes isolated from tumors.

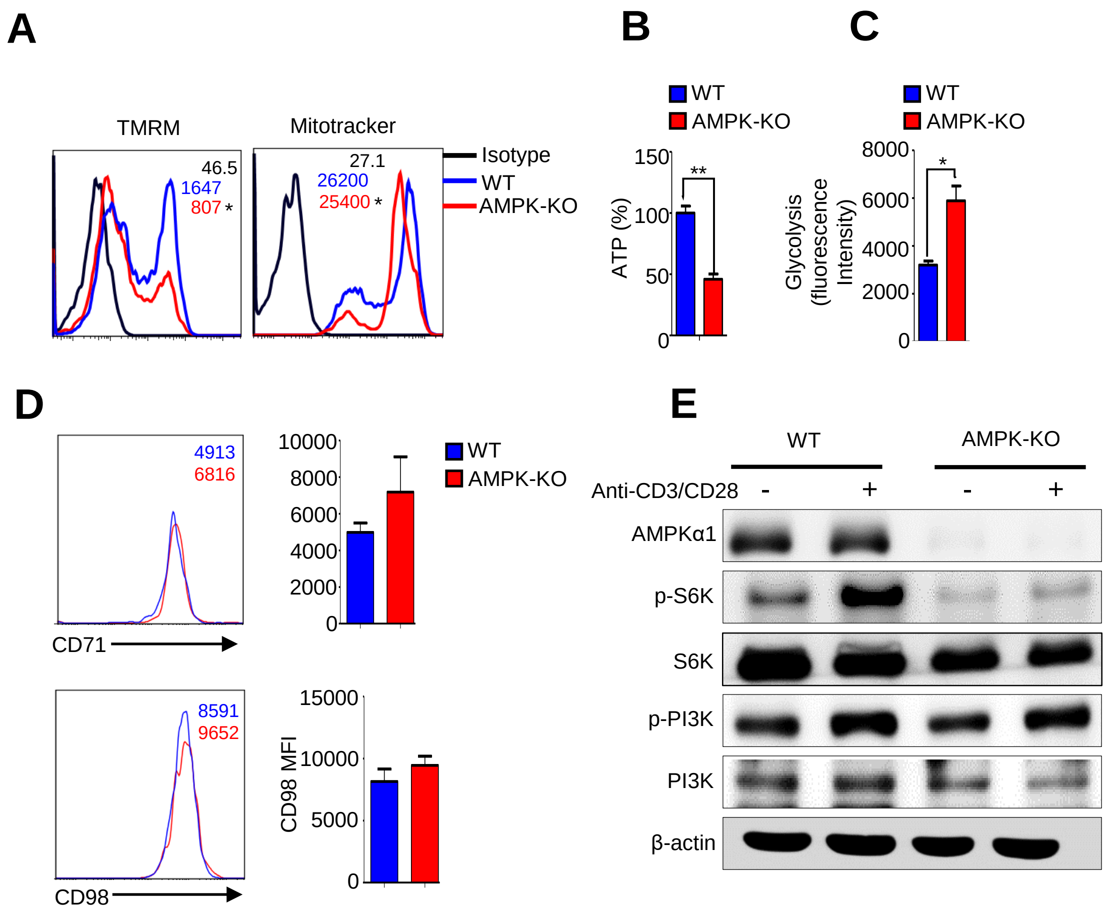

Figure S11. Deficiency of AMPK in Tregs changes metabolic process with independent of mTORC1 signaling (A) Flow cytometric analysis of splenic Tregs stained with TMRM and Mitotracker. **(B)** Comparison of ATP production in WT and AMPK-KO Tregs. **(C)** Fluorescence intensity of the glycolysis assay signal measured at the excitation and emission wavelengths of 380 and 615 nm, respectively. **(D)** MFI of CD71 and CD98 in Tregs isolated from B16F10 tumor tissues from WT and AMPK-KO mice after 4 h of stimulation with anti-CD3/CD28 antibodies. (E) Western Blot analysis of phosphorylated and total S6K and PI3K in WT and AMPK-KO Tregs stimulated with or without anti-CD3/CD28 stimulation for 4 h.

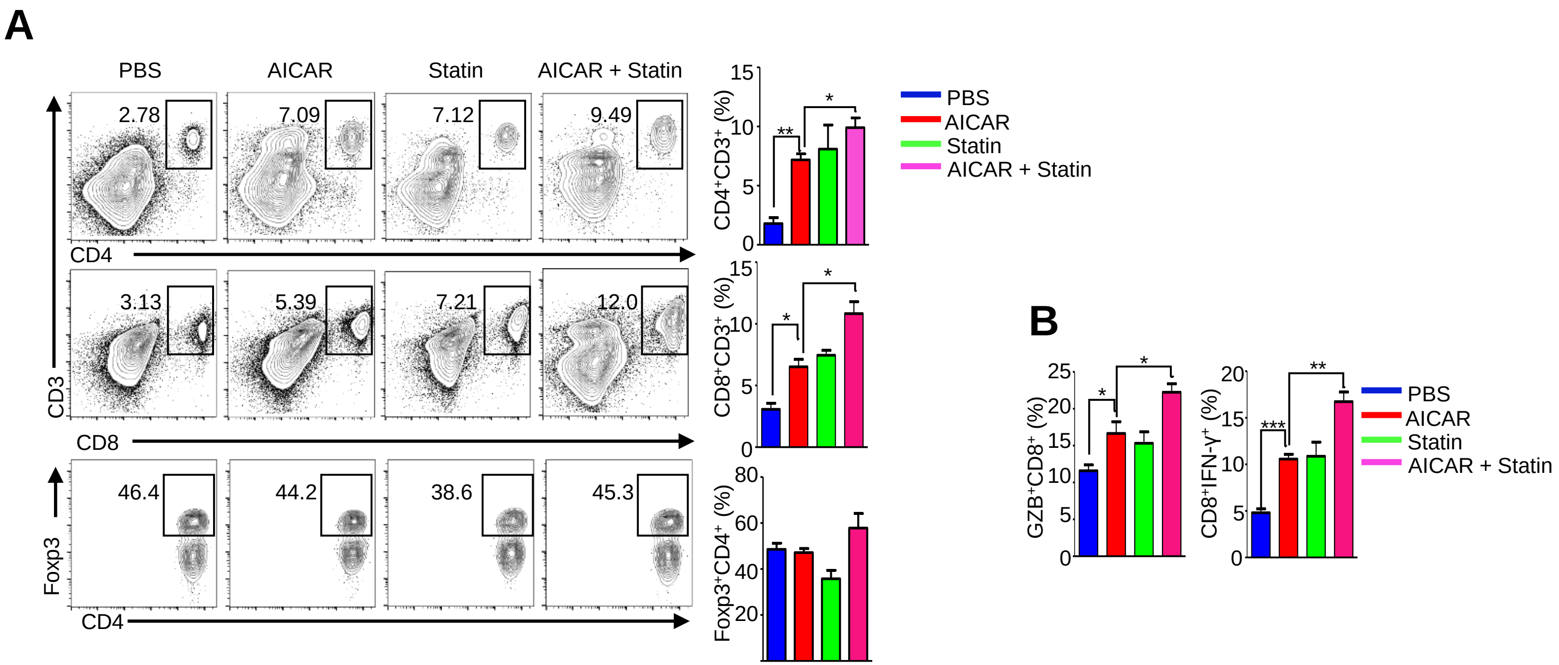

Figure S12. Combined therapy of AICAR and Statin increases effector T cells. (A) Flow cytometric analysis of the percentage of $CD4^+$ and $CD8^+$ T cells and $CD4^+Foxp3^+$ Tregs in tumors from WT mice treated with PBS, AICAR, statin, or the combination. **(B)** Bar diagram representation of flow cytometric analysis of percentage of GZB- and IFN-γ-producing $CD8^+$ T cells in B16F10 tumors from WT mice treated with PBS, AICAR, statin, or the combination, n=5 mice per group.

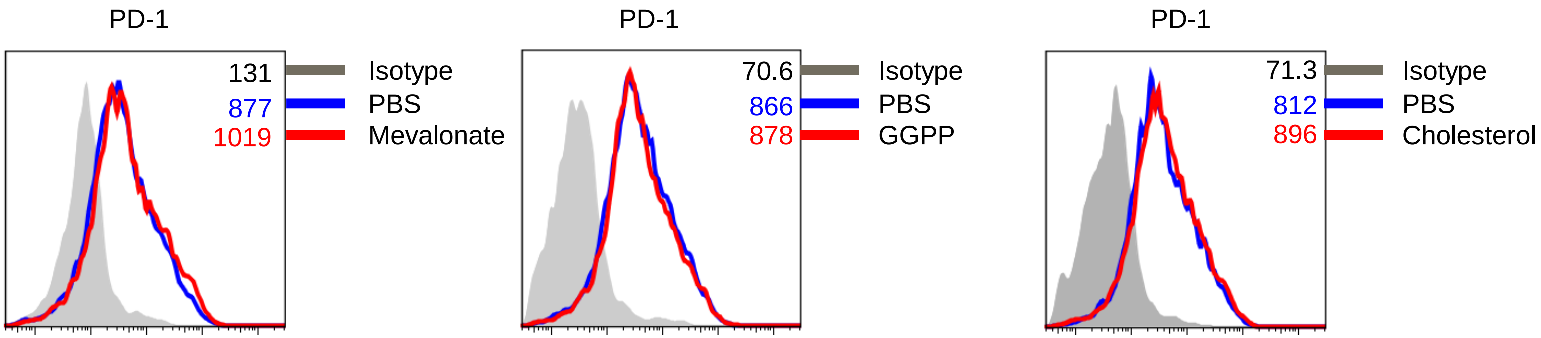

Figure S13. PD-1 expression in splenic Tregs from WT mice treated with mevalonate pathway byproducts *in vivo*. Flow cytometry analysis and MFI of PD-1 expression in splenic Tregs treated with mevalonate, GGPP, and cholesterol for 24 h, n=5 mice per group.
